# VCP Phosphorylation-Dependent Interaction Partners Prevent Apoptosis in *Helicobacter pylori*-Infected Gastric Epithelial Cells

**DOI:** 10.1371/journal.pone.0055724

**Published:** 2013-01-31

**Authors:** Cheng-Chou Yu, Jyh-Chin Yang, Yen-Ching Chang, Jiing-Guang Chuang, Chung-Wu Lin, Ming-Shiang Wu, Lu-Ping Chow

**Affiliations:** 1 Graduate Institute of Biochemistry and Molecular Biology, College of Medicine, National Taiwan University Hospital, Taipei, Taiwan; 2 Department of Internal Medicine, National Taiwan University Hospital, Taipei, Taiwan; 3 Department of Pathology, National Taiwan University Hospital, Taipei, Taiwan; Veterans Affairs Medical Center (111D), United States of America

## Abstract

Previous studies have demonstrated that valosin-containing protein (VCP) is associated with *H. pylori*-induced gastric carcinogenesis. By identifying the interactome of VCP overexpressed in AGS cells using a subtractive proteomics approach, we aimed to characterize the cellular responses mediated by VCP and its functional roles in *H. pylori*-associated gastric cancer. VCP immunoprecipitations followed by proteomic analysis identified 288 putative interacting proteins, 18 VCP-binding proteins belonged to the PI3K/Akt signaling pathway. *H. pylori* infection increased the interaction between Akt and VCP, Akt-dependent phosphorylation of VCP, levels of ubiquitinated proteins, and aggresome formation in AGS cells. Furthermore, phosphorylated VCP co-localized with the aggresome, bound ubiquitinated proteins, and increased the degradation of cellular regulators to protect *H. pylori-*infected AGS cells from apoptosis. Our study demonstrates that VCP phosphorylation following *H. pylori* infection promotes both gastric epithelial cell survival, mediated by the PI3K/Akt pathway, and the degradation of cellular regulators. These findings provide novel insights into the mechanisms of *H. pylori* infection induced gastric carcinogenesis.

## Introduction

Gastric cancer is the second most common cause of death from cancer worldwide [1]. While a number of factors probably influence an individual's predisposition to gastric cancer, evidence is increasing that gastric cancer originates from a chronic inflammatory process [2], [3]. *Helicobacter pylori* (*H. pylori*) infects 50% of the world's population and is now known to be responsible for inducing chronic gastric inflammation that progresses to atrophy, metaplasia, dysplasia, and gastric cancer. The association between chronic *H. pylori* infection and the development of gastric cancer is well established. Chronic inflammation promotes apoptosis, which can lead to a compensatory proliferative response by the remaining tissues [4]. The dynamic balance between cell proliferation and apoptosis is important in maintaining mucosa homeostasis and, when the balance is disturbed by infection, injury, or cytokines, decreased apoptosis and increased proliferation might favor the carcinogenesis [5]. Thus, understanding the mechanisms by which *H. pylori* regulates the fate of cells is critical for establishing potential therapeutic approaches to prevent *H. pylori* infection-induced gastric carcinogenesis.

A previous comparative proteomic approach demonstrated increased levels of many cancer-related factors in *H. pylori*-infected AGS cells [6]. One of these was valosin-containing protein (VCP), a type II AAA (ATPases associated with various cellular activities), which has a stable ring structure consisting of homo-hexamers [7] and contains two copies of the conserved ATP-binding domain and an N-terminal polyubiquitin-binding domain [8] and can associate with large complexes containing ubiquitinated proteins and transport the complexes to the aggresome [8]–[10]. Aggresome formation is a general response of cells that occurs when the capacity of the proteasome is exceeded by the production of aggregation-prone misfolded proteins. VCP acts as a molecular chaperone and is implicated in a large number of ATP-dependent cellular processes, such as membrane fusion, ubiquitin/proteasome-mediated proteolysis, endoplasmic reticulum-associated degradation, transcription activation, the stress response, cell cycle regulation, and apoptosis [10]–[15]. The aggresome pathway and VCP inhibitors are emerging fields in cancer therapy.

VCP is upregulated after *H. pylori* infection [6], and several lines of evidence indicate that its level of expression is highly associated with tumor progression and prognosis in non-small cell lung cancer, hepatocellular carcinoma, pancreatic ductal adenocarcinoma, and gastric carcinoma [16]–[19]. This correlation suggests that the level of VCP expression could be used as potential marker for the progression of these cancers [20]–[22]. However, the molecular function of VCP in *H. pylori*-infected gastric epithelial cells is still unclear. Since protein-protein interactions are crucial for most biological processes, a systemic identification of VCP-interacting proteins might provide new clues about the role of VCP activation in the pathogenesis of *H. pylori* infection in gastric epithelial cells.

In this study, we used a subtractive proteomics-based approach to identify VCP-interacting proteins to explore the VCP signaling pathway. Proteins co-immunoprecipitated with VCP were analyzed by mass spectrometry and a series of putative physically interacting partners were defined. Furthermore, transactivation of VCP by *H. pylori* protected gastric epithelial cells from apoptosis and this anti-apoptotic response was mediated by phosphatidylinositol 3-kinase (PI3K)-dependent activation of Akt and degradation of cellular regulators. These results will be important in further investigations of the mechanisms of *H. pylori*-associated carcinogenesis.

## Materials and Methods

### Cell culture, adenoviral transduction, and transient transfection

AGS human gastric epithelial cells and human embryonic kidney 293 cells (HEK 293) were grown in Dulbecco's modified Eagle's medium (DMEM) containing 10% fetal bovine serum (FBS) (both from Invitrogen Corp., Carlsbad, Calif., USA), 2 mM glutamine, and 100 units/ml of penicillin-streptomycin at 37°C in 5% CO2. Adenovirus particles coding for VCP-Flag or empty vector were propagated, purified, and titrated in HEK 293 cells. For adenoviral transduction, AGS cells were infected with adenovirus particles coding for VCP-Flag or empty vector at a multiplicity of infection (moi) of 10–50, then the transduced cells were cultured in DMEM for 36 hr. In addition, AGS cells were transiently transfected with various DNA constructs including wild type VCP, mutant VCP (Material and Methods S1) or short-hairpin RNA (shRNA) using Lipofectamine 2000 (Invitrogen) according to the manufacturer's instructions, then the transfected cells were cultured in fresh medium containing 10% FBS for 16–18 hr before use.

### Bacterial Strain and Growth Conditions

The *H. pylori* strain (TA1) is CagA positive and produces VacA vacuolating cytotoxin. It was used in this study was isolated from human gastric biopsy samples obtained from patients with gastric cancer at the National Taiwan University Hospital, Taipei, Taiwan. The bacteria were inoculated onto Columbia agar containing 5% sheep blood (Invitrogen) and grown at 37°C in a microaerophilic chamber (Don Whitley, West Yorkshire, UK) in 10% CO_2_, 5% O_2_, and 85% N_2_.

### Cell treatment and sample preparation

For coculture of *H. pylori* and AGS cells, the bacteria were washed off the plates and resuspended in PBS to an OD at 450 nm of 1.0 units, corresponding to a bacterial concentration of 2×10^11^ CFU/L, and added to wells containing 2×10^5^ gastric epithelial cells at an *H. pylori*∶AGS cell ratio of 100∶1 and were then cocultured in a cell culture incubator. Cells were pretreated with 10 µM LY294002 or 10 µM MG-132 for 3 hr before infection for 6 hr with *H. pylori* at an moi of 100, then were lysed with lysis buffer (25 mM Tris, pH 7.5, 1 mM MgCl_2_, 1 mM EGTA, 150 mM NaCl, 1% v/v NP-40) containing 1% v/v protease and phosphatase inhibitor cocktails and the lysates collected using a cell scraper and centrifuged at 1,200 *g*, 4°C for 15 min, then the protein concentration of the supernatant was determined using a BCA™ protein assay kit.

### Immunoprecipitation and immunoblotting

To examine the VCP-interacting proteins, cells overexpressing VCP-Flag or empty vector that were incubated with *H. pylori* were lysed with lysis buffer containing protease inhibitors and the lysate centrifuged at 1,200 *g*, 4°C for 15 min, then the cell extract were immunoprecipitated for 4 hr at 4°C with anti-Flag M2 affinity gel and the immunoprecipitates analyzed by immunoblotting to identify interacting proteins.

Protein extracts (20 µg/lane) were separated by 10% SDS PAGE electrophoresis and transferred to a PVDF membrane, which was then blocked for 1 hr at 25°C with 5% nonfat dry milk in Tris-buffered saline, pH 7.5 (TBS). The blot was then incubated overnight at 4°C with rabbit antibodies against p-Akt substrate (PAS), Akt, pAkt, p53, p27, p21, programmed cell death 4 (PDCD4), Caspase 9, Bcl-2-associated death promoter (BAD), or p-IκBα or mouse antibodies against ubiquitin. Rabbit antibodies against β-GAPDH were used to provide the loading control. After six washing steps, the membranes were incubated for 1 hr at 25°C with horseradish peroxidase (HRP)-conjugated anti-rabbit IgG or anti-mouse IgG antibodies, then, after another six washing steps, bound antibodies were detected using ECL reagent. Densitometry was performed using ImageQuantv.5.2 software from Molecular Dynamics (Amersham Biosciences).

### BALB/c mice infection and immunohistochemistry

Five-week-old male specific pathogen-free BALB/c mice were used and housed at the Experimental Animal Center, National Taiwan University, at a constant temperature. All surgical and experimental procedures were approved by the Institutional Animal Care and Use Committee of the National Taiwan University College of Medicine (Taipei, Taiwan, approval ID = 20100002).

The *H. pylori* infection mouse model was modified from that in a previous study [23], [24]. A set of 22 samples from a cohort of 30 BALB/c mice were used in this study. The *H. pylori* strain TA1 was used to intragastric (i.g.) infect mice. 20 mice were inoculated i.g. 2 times on successive days with 0.5 mL (10^11^ bacteria/L) of bacterial suspension. Uninfected control mice received distilled water only. Four weeks after *H. pylori* inoculation, the mice were killed by anesthesia with 0.2–0.5 ml of 50% urethane and their stomachs removed and longitudinally divided into 2 equal parts for histological and microbiological examination. *H. pylori* was positively identified after 3–5 day culture and the CFU of *H. pylori* counted after culturing. Gastritis was graded by the pathologist without knowledge of the treatment protocol according to the updated Sydney system [24], [25]. Confirmation of *H. pylori* status in gastric tissue was adapted by PCR [24]–[26]. *H. pylori* infection causes gross mucosal injury, including edema, hemorrhage, and inflammation in mice by pathological analysis [24]. At necropsy, linear strips extending from the squamocolumnar junction to the proximal duodenum were fixed in 10% neutral-buffered formalin, paraffin-embedded, and stained with hematoxylin and eosin stain or with anti-VCP antibody (1∶200), then the immunohistochemistry for VCP detection was performed on paraffin-embedded tissue sections as described previously [6]. Stained sections were evaluated without prior knowledge of the clinicopathologic parameters. Staining intensity was categorized as low (scored as 1) or high (scored as 2 or 3).

### Purification of VCP-interacting proteins

Cells were infected with adenovirus particles coding for VCP-Flag or empty vector, pretreated with 10 µM MG-132 for 3 hr before infection for 6 hr with *H. pylori*, and lysed with lysis buffer containing 1% v/v proteinase and phosphatase inhibitor cocktail. To immunoprecipitate Flag-tagged proteins, the cell lysates were mixed overnight at 4°C with anti-Flag M2 affinity gel, then the gel was poured into a column, washed three times with lysis buffer, and eluted with sample buffer. The eluted proteins were separated by 10% SDS/PAGE and examined by immunoblotting.

### In gel digestion, mass spectrometry analysis, and protein identification

After silver staining, individual protein bands were excised from the gel and digested overnight at 37°C with trypsin (1 µg/µl; Promega), then the tryptic peptide mixture was eluted for 30 min at 4°C from the gel with 100 µl of 0.1% TFA, followed by 100 µl of 0.1% TFA/60% acetonitrile, and the combined extracts lyophilized. The tryptic peptides were resuspended in 0.1% TFA and analyzed by LC-MS/MS using an LTQ-Orbitrap Velos hybrid mass spectrometer (Thermofisher Scientific, Waltham, MA). Peptide separations were performed on-line with MS by nanoflow liquid chromatography (Dionex Ultimate 3000 Dionex Corporation, CA). Samples were injected in a 10 µL volume of starting mobile phase (2% acetonitrile in 0.1% formic acid) at a flow rate of 750 nL/min onto a Nanoacquity LC system with a 75 um×15 cm C18 column. Samples were loaded for 15 min before switching the sample loop out of the flow path and decreasing the flow to 300 nL/min. The peptides were then separated by elution with a linear gradient of 2–90% acetonitrile in 0.1% formic acid at 300 nL/min in 90 min and injected onto a Thermo Scientific Velos Orbitrap electrospray mass spectrometer running at a resolution of 60 000 for precursors. Data-dependent MS/MS analysis was carried out using Xcalibur MS acquisition software (Xcalibur 2.1, ThermoFisher Scientific). Each scan cycle consisted of a full scan MS acquired in profile mode at 60K resolution by the Orbitrap analyzer over the mass range 350–1600 m/z, followed by data-dependent MS/MS scans of the 20 most intense peaks. MS/MS spectra were acquired in higher-energy collisional dissociation mode with 35% normalized collision energy. Dynamic exclusion was set for 90 s with a 20 ppm window and monoisotopic precursor selection was enabled. Proteins were identified by Mascot search engine (version 2.2.2, Matrix Science) against Swiss-Prot version 56 protein database. The search parameters were set as follows: Carbamidomethylation (C) and oxidation (M) as variable modifications, 0.3 Da for MS tolerance, 0.5 Da for MS/MS tolerance, and 2 for missing cleavage. The individual score for the MS/MS spectrum of each peptide was more than 20.

### VCP-interacting protein network pathway analysis

A list of VCP-interacting proteins was uploaded to the Ingenuity Pathway Analysis (IPA) software to investigate the biological networks associated with these proteins (http://www.ingenuity.com). This analysis uses computational algorithms to identify networks consisting of focus proteins (proteins that were present in our list) and their interactions with other proteins (“non-focused”) in the knowledge base. Scores were calculated for each network according to the fit of the network to the set of focus proteins and used to rank networks in the Ingenuity analysis. IPA uses the proteins from the highest-scoring network to extract a connectivity pathway that relates candidate proteins to each other based on their interactions. These candidates were shown to be significantly associated with the Function, Disease, and Canonical Pathways. In addition, we also searched for other proteins involved in this network using the build tools of IPA. Significance of the biofunctions and the canonical pathways were tested by the Fisher Exact test p-value [27].

### Assessment of apoptosis

Cells pretreated with 10 µM LY294002 for 3 hr or transfected with shRNAs, wild-type VCP, or a triple Ser mutant of VCP (Tri-mut VCP) were incubated with *H. pylori* for 24 hr, then were stained for 10 min at room temperature with Annexin V-FITC and propidium iodide (PI) [28] and the percentage of apoptotic cells measured using a Cytomics FC500 flow cytometer (Beckman-Coulter, Villepinte, France).

### Cell viability assay

Cell viability was assessed using an MTS kit (Promega) using the manufacturer's protocol. Briefly, AGS cells in 96-well plates were transfected with VCP-Flag or Tri-mut VCP-Flag plasmids for 24 hr, then were incubated with or without *H. pylori* for 24 hr. MTS reagent was then added to each well and the plates incubated at 37°C for 1 hr, when the absorbance at 490 nm was measured. The OD_490_ reading for VCP-Flag-transfected cells not incubated with *H. pylori* was taken as the 100% value. The experiment was performed five times.

### Cell cycle analysis

AGS cells were transfected with VCP-Flag and Tri-mut VCP-Flag plasmids for 24 hr, then incubated with or without *H. pylori* for 24 hr, rinsed with phosphate-buffered saline (PBS), and fixed overnight in ice-cold ethanol (70% v/v). The fixed cells were then washed twice with by PBS and re-suspended in staining solution containing 10 mg/ml of PI (Sigma), 20 mg/ml of RNAse A (Sigma), and 0.1% BSA. After incubation for 1 hr at room temperature, DNA content was determined using a Cytomics FC500 flow cytometer. Cells with a DNA content between 2 n and 4 n, designated as being in the G0/G1 phase of the cell-cycle, were counted using Cell Quest software.

### Immunofluorescence analysis

AGS cells transfected with wild type VCP-Flag or Tri-mut VCP-Flag expression constructs were fixed with cold methanol for 10 min, then immunostained with antibodies against VCP, ubiquitin, p53, p27, p21, Caspase 9, BAD, or PDCD4, followed by secondary antibodies conjugated to FITC or TRITC. Aggresomes were detected using a ProteoStat kit and nuclei stained with DAPI. Cell images were captured using a TCS SP5 confocal microscope (Leica).

### Electron microscopy

In situ cell fixation and processing were performed as described previously [29]. Briefly, AGS cells cultured on a 6 cm culture plate were fixed with 2% glutaraldehyde followed by 1% osmium tetraoxide buffered in 0.1 M sodium cacodylate (pH 7.4). The cells were then *en bloc* stained with 0.5% uranyl acetate aqueous solution and dehydrated in a series of ethanol, embedded in epoxy resin, and cured in a 55°C oven for 48 hr. Thin sections were then mounted on 300 mesh grids, stained with uranyl acetate and lead citrate, and stabilized by carbon evaporation. The sections were examined and photographed on an H7100 electron microscope (Hitachi).

### Statistical analysis

Experiments for the detection of immunoblotting, apoptosis, cell viability, and cell cycle were performed at least three times. The paired *t* test was used for statistical analysis between two groups. Significant level was set at *p<0.05, **p<0.01. Values for all measurements were expressed as the mean ±SD.

## Results

### Immunohistochemical studies

**Figure 1:**
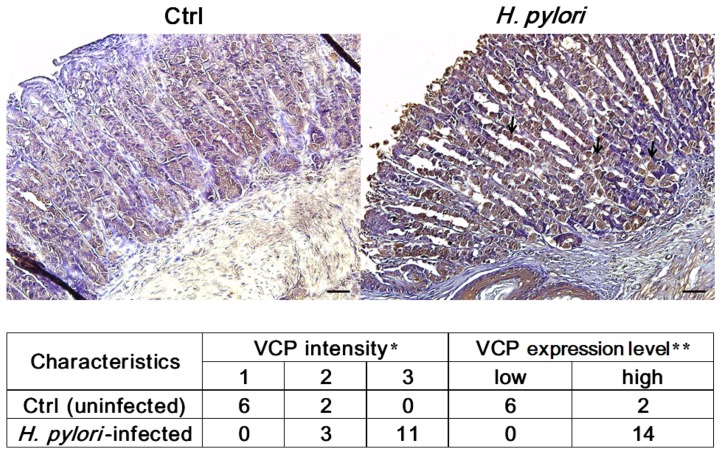
*H. pylori* infection induced high expression of VCP in mouse gastric tissue. Upper panel: Representative VCP staining (200×) in an uninfected and an infected mouse at 2 weeks after infection. The dark brown color indicates VCP high expression (arrow). Scale bar, 50 µm. Lower panel: Summary of the immunohistochemical data for the correlation between VCP expression and *H. pylori*-infected mouse gastric tissue. A total number of 22 mouse gastric tissue, including uninfected (n = 8) and *H. pylori*-infected (n = 14) mouse gastric tissues, were analyzed for VCP expression. *VCP intensity by immunohistochemistry staining was scored as following: 1, weak staining; 2, mild staining and 3, dark staining. **Based on the score of VCP intensity, cases were classified into VCP low-expression (low) for those cases in which of VCP intensity scores were ≤1 and VCP high-expression (high) for those cases in which of VCP intensity scores were ≥2.

We previously showed that, in 9 out of 10 *H. pylori*-infected patients, staining for VCP was stronger in gastric cancerous tissues than in the surrounding normal tissues [6]. To clarify the correlation between VCP expression and *H. pylori*-infected gastric cancer, we also assessed VCP expression level *in vivo* using a set of 22 samples from a cohort of 30 BALB/c mice (Figure 1). The samples we analyzed were from uninfected (n = 8) and *H. pylori*-infected BALB/c mice (n = 14). We found that most uninfected controls (n = 6) showed low expression of VCP, whereas all *H. pylori*-infected BALB/c mice showed high expression of VCP (n = 14). Taken together, immunohistochemistry of *H. pylori*-infected BALB/c mice showed that VCP was high expression compared to uninfected controls (Figure 1). These results suggested that VCP expression is correlated with the risk of gastric cancer under *H. pylori* infection.

### Identification of VCP-interacting protein complexes

**Figure 2:**
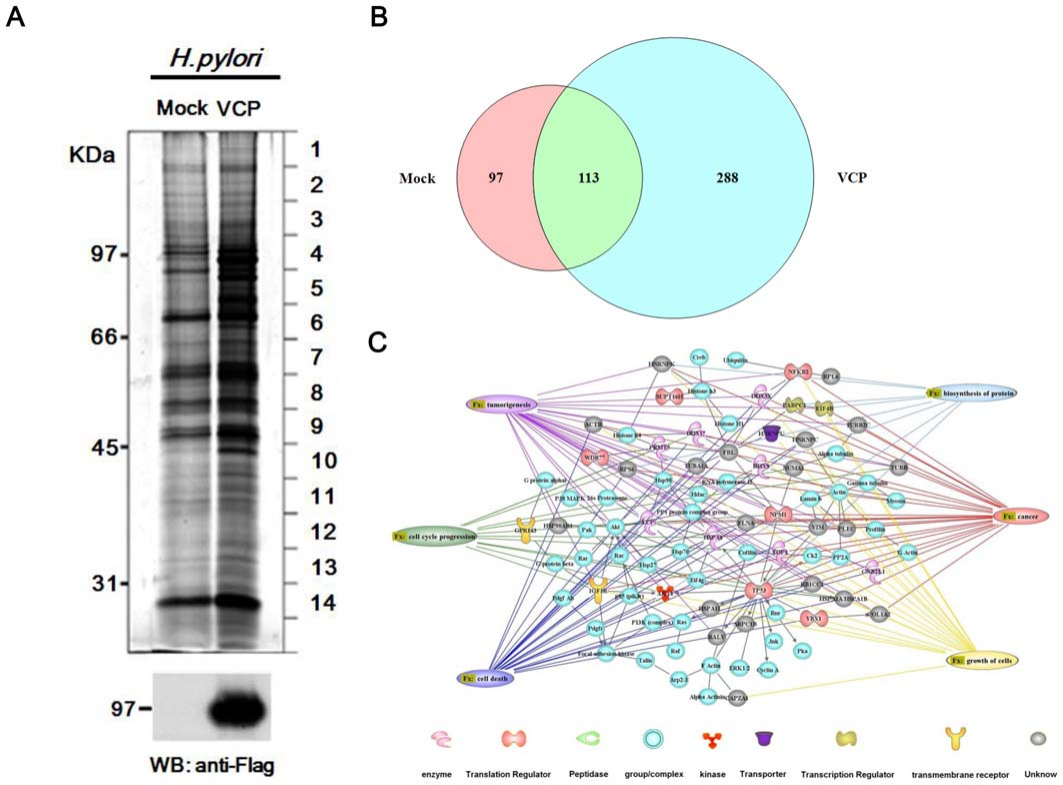
Ingenuity Pathway Analysis (IPA) prediction and analysis of signal transduction networks for the 288 proteins in the VCP interactome in AGS cells. *(A)* AGS cells overexpressing VCP-Flag or mock infected were pretreated 10 µM MG-132 for 3 hr and co-cultured with *H. pylori* for 6 hr, then immunoprecipitation was performed using anti-Flag antibody affinity gel and the precipitated proteins examined on silver-stained SDS gels (top panel) and immunoblots using anti-Flag antibodies (bottom panel). The lines on the right of the gel indicate where the gel was cut and the sections digested with trypsin and the peptides analyzed by mass spectrometry analysis; the identified VCP-binding proteins are listed in Table S2. *(B)* Proportionately drawn Venn diagram analysis of the total proteins in Mock and VCP co-immunoprecipitates. *(C)* The networks were generated by the shortest path algorithm of the Ingenuity Pathway Analysis (IPA) software using the list of 288 proteins in VCP co-immunoprecipitates identified by LC-MS/MS analysis (Table S2). The lines connecting molecules indicate molecular relationships.

To investigate the functional roles of VCP involving the carcinogenic effect of *H. pylori* on the gastric epithelial cell. We used a subtractive proteomic approach to identify proteins that physically interact with VCP, but not with empty vector that are expressed in response to *H. pylori* infection. AGS cells were infected with adenovirus carrying the expression vector for VCP-Flag or empty vector for 36 hr, and treated with *H. pylori*, then lysates of the cells were immunoprecipitated with anti-Flag M2 affinity gel and the eluted proteins separated by SDS-PAGE and immunoblotting with anti-FLAG antibody. As shown in Figure 2A, multiple protein bands, which might be VCP-binding proteins, were seen in *H. pylori*-treated lysates compared to the control. The stained SDS gel was separated into 14 equal parts (Figure 2A), which were subjected to in gel tryptic digestion and the peptides generated analyzed by LTQ-Orbitrap mass spectrometry. Through comparative analyses, 97 were identified only in control co-immunoprecipitates, 113 proteins were identified in both co-immunoprecipitates, and 288 were identified only in VCP co-immunoprecipitates (Figure 2B); the names of these 288 proteins, their accession numbers in the Swiss-Prot database, molecular mass, Mascot score, and percentage of coverage are listed in Table S2.

### Organization of the VCP-interacting proteins and mapping of possible biological signal transduction networks by Ingenuity Pathway Analysis (IPA)

Next, using Ingenuity Pathway Analysis (IPA) software, we determined whether the 288 VCP-binding proteins could be grouped into different functional classes. The results are illustrated in Figure 2C and the details are presented in Table S3. 87 of which were grouped into different functional classes and the function to which the highest number of binding proteins belonged were cancer (*n* = 47), growth of cells (*n* = 35), tumorigenesis (*n* = 34), death of cancer cells (*n* = 34), cell cycle progression (*n* = 30), biosynthesis of protein (*n* = 13), and tumorigenesis (*n* = 34). To determine which of the pathway contained the most crucial activity, our analysis also highlighted a number of canonical pathways, which included PI3K/Akt signaling, the protein ubiquitination pathway, aldosterone signaling in epithelial cells, glycolysis/gluconeogenesis, p70S6K signaling, glucocorticoid receptor signaling, mitotic roles of Polo-Like kinase, hypoxia signaling in the cardiovascular system, ERK/MAPK signaling, and 14-3-3 protein-mediated signaling (Table 1). The analysis indicated that multiple VCP-binding proteins belonged to the PI3K/Akt signaling pathway (*n* = 18), suggesting that Akt interacts with VCP and controls this pathway in *H. pylori*-infected AGS cells.

**Table 1:** Top 10 list of canonical pathways activated by VCP-interacting proteins from Ingenuity Pathway Analysis.

Ingenuity Canonical Pathways	#of gene	Molecules	−log(p-value)
PI3K/Akt Signaling	18	AKT1, BAX, ERK1/2, Hsp90, HSP90AB1, MDM2, mTOR, NFKB2, PDCD4, P38 MAPK, p85 (pik3r), PI3K (complex), PP2A, Raf, Ras, TP53, 14-3-3 protein	5.89E00
Protein ubiquitination pathway	9	26 s Proteasome, Hsp27, Hsp70, Hsp90, HSP90AB1, HSPA8, HSPA1A/HSPA1B, HSPA1L, Ubiquitin	5.3E00
Aldosterone signaling in epithelial cells	13	ERK1/2, Hsp27, Hsp70, Hsp90, HSP90AB1, HSPA8, HSPA1A/HSPA1B, HSPA1L, P38 MAPK, p85 (pik3r), PI3K (complex), Raf, Ras	4.71E00
Glycolysis/gluconeogenesis	9	AKT1, ERK1/2, G-protein beta, GNB2L1, P38 MAPK, p85 (pik3r), PI3K (complex), Raf, Ras	4.51E00
p70S6K signaling	10	AKT1, ERK1/2, G protein alphai, P38 MAPK, p85 (pik3r), PI3K (complex), PP2A, Raf, Ras, RPS6	3.9E00
Glucocorticoid receptor signaling	14	AKT1, Histone h3, Hsp70, Hsp90, HSP90AB1, HSPA8, HSPA1A/HSPA1B, HSPA1L, Jnk, P38 MAPK, p85 (pik3r), PI3K (complex), Pka, Rac	3.62E00
Mitotic roles of Polo-like kinase	6	AKT1, Jnk, P38 MAPK, p85 (pik3r), PI3K (complex), Ras	3.55E00
Hypoxia signaling in the cardiovascular system	6	AKT1, Creb, Hsp90, HSP90AB1, PI3K (complex), TP53	3.41E00
ERK/MAPK signaling	5	Creb, Histone h3, Hsp27, Jnk, P38 MAPK	3.16E00
14-3-3 protein-mediated signaling	8	AKT1, Alpha tubulin, ERK1/2, Gamma tubulin, TUBA1A, TUBB, TUBB2C, VIM	2.71E00

### Validation of the VCP-interacting proteins and stimulation of VCP phosphorylation in *H. pylori*-infected AGS cells

**Figure 3:**
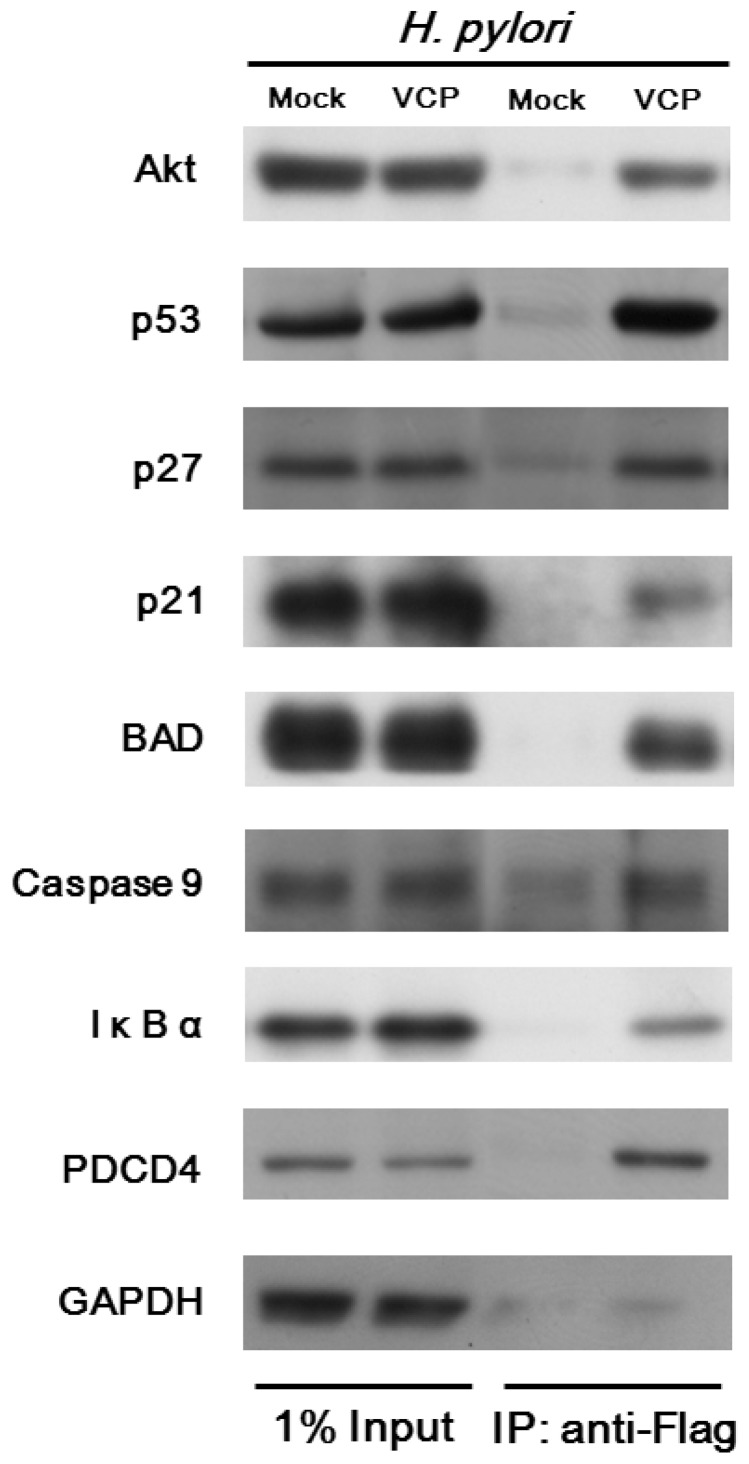
Immunoprecipitation and immunoblotting analysis of VCP interaction partners in *H. pylori*-infected AGS cells. AGS Cells were transfected with plasmid coding for VCP-Flag or empty vector, pretreated with MG-132 for 3 hr, and incubated for 6 hr with *H. pylori*. Immunoprecipitations were performed with anti-Flag M2 antibody affinity gel and the immunoprecipitates analyzed by immunoblotting to identify VCP interaction partners. 1% of input was subjected to immunoblotting.

We next determined whether the VCP-interacting proteins belonging to the Akt pathway noted in the IPA analysis were functionally relevant. According to the bioinformatics analysis and literature review for VCP-interacting proteins identified in the present study showed that many of these proteins were involved in PI3K/Akt signaling. The proteins selected were IκBα, Caspase9, BAD, PDCD4, p53, p21^Cip1^, and p27^kip1^, which are involved in NF-κB activation, cell survival, cell death and cell cycle progression (Figure S1). To verify the bioinformatics and immunoprecipitation results, we performed an immunoblotting analysis on above selected protein targets. As shown in Figure 3, these data confirm that the selected protein targets were VCP binding proteins. Because Akt activation which plays a pivotal role in *H. pylori*-infected AGS cells [28], [30], [31], we then examined whether phosphorylation of Akt affected the interaction between VCP and Akt in *H. pylori*-infected AGS cells. As shown in Figure S2A, *H. pylori* infection stimulated both activation of Akt and VCP phosphorylation, as shown using anti-phospho (Ser/Thr)-Akt substrate (PAS) antibody, which recognizes motifs phosphorylated by Akt, and pretreatment with the PI3K inhibitor LY294002, which inhibits activation of Akt, caused reduced levels of VCP phosphorylation in *H. pylori*-infected cells. These data show that *H. pylori* infection of AGS cells stimulates VCP phosphorylation through activation of Akt.

### Identification of sites on VCP for *H. pylori*-induced phosphorylation

Having demonstrated PI3K-dependent stimulation of Akt phosphorylation and the VCP-Akt interaction and that VCP phosphorylation sites were recognized by anti-PAS antibody in *H. pylori*-infected AGS cells, we tested our hypothesis that VCP was a target of Akt kinase-mediated signaling. We performed a bioinformatics search using Scansite software to identify putative sites in VCP that could be phosphorylated by Akt (consensus site defined as RXRXX(S/T) [32]) and identified three possible sites (AATNRPNS**352**, AMRFARRS**746**, and RFARRSVS**748**) (Figure S2B). To test the involvement of Ser-352, Ser-746, and Ser-748, we performed site-directed mutagenesis and generated plasmids coding for the single mutants VCP-Flag^S352A^, VCP-Flag^S746A^, and VCP-Flag^S748A^, the double mutants VCP-Flag^S352A/S746A^, VCP-Flag^S352A/S748A^, and VCP-Flag^S746A/S748A^, and the triple mutant VCP-Flag^S352AS746AS748A^ (Tri-mut VCP-Flag). The VCP wild-type and mutant plasmids were used to transfect AGS cells, which were then incubated with or without *H. pylori*, then the lysates were immunoprecipitated with anti-Flag antibody and phosphorylation of the Akt substrate phosphorylation consensus sites detected using anti-PAS antibody. As shown in Figure S2C, after *H. pylori* infection, wild-type VCP was phosphorylated to a greater extent than single mutants S352A, S746A, and S748A, while the double mutants showed even less phosphorylation and the triple mutant S352AS746AS748A was not phosphorylated. These results show that Ser-352, Ser-746, and Ser-748 are sites for Akt-mediated phosphorylation of VCP.

### VCP is functionally involved in cell survival through activation of Akt

**Figure 4:**
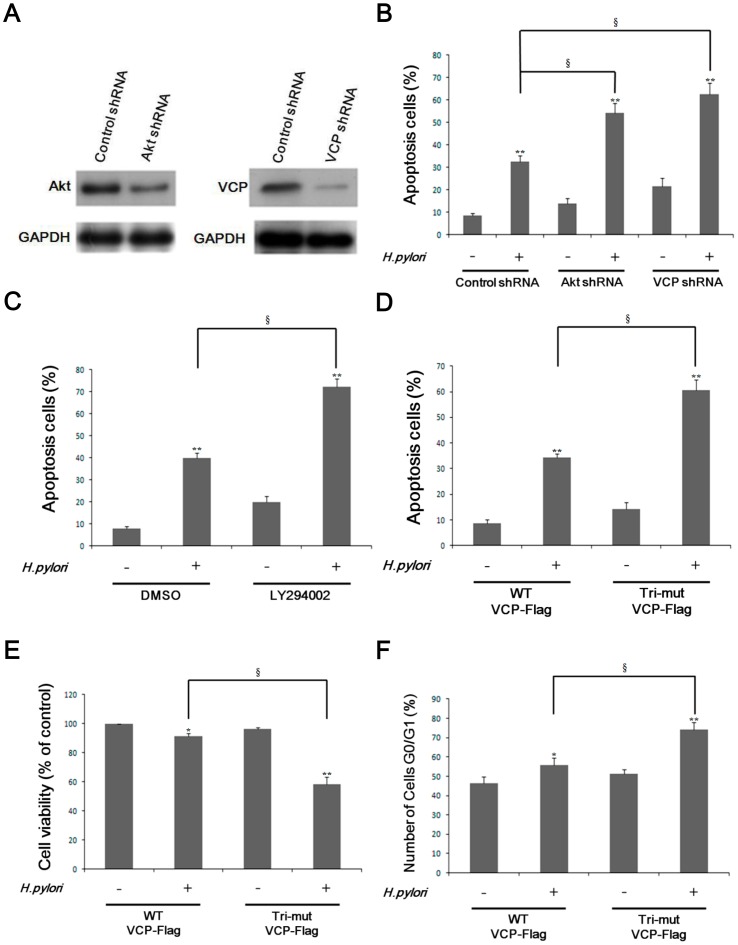
Akt activity is involved in VCP phosphorylation-induced protection against apoptosis and proliferation and cell division of AGS cells infected with *H. pylori*. *(A)* AGS cells were transfected with control shRNA, Akt shRNA, or VCP shRNA for 36 hr, then were analyzed by immunoblotting. GAPDH served as the loading control. *(B–D)* Cells were stained with Annexin V/propidium iodide(PI) and the percentage of apoptotic cells analyzed by flow cytometry. *(B)* Apoptosis of AGS cells transfected with control shRNA, Akt shRNA, or VCP shRNA with or without *H. pylori* infection for 24 hr. **p<0.01 relative to cells uninfected with *H. pylori*. **§** *p*<0.01 relative to cells transfected with control shRNA. Data are expressed as means ± SD. *(C)* Apoptosis of AGS cells with or without pretreatment with 10 µM LY294002 for 3 hr with or without *H. pylori* infection for 24 hr. **p<0.01 relative to cells uninfected with *H. pylori*. **§** *p*<0.01 relative to cells treated with DMSO. Data are expressed as means ± SD. *(D)* Apoptosis of AGS cells transfected withVCP-Flag or Tri-mut VCP-Flag with or without *H. pylori* infection for 24 hr. *(E)* Proliferation of AGS cells transfected with VCP-Flag or Tri-mut VCP-Flag with or without *H. pylori* infection for 24 hr. Cell proliferation was measured using the MTS assay. *(F)* Percentage of cells in G0/G1 phase in AGS cells transfected with VCP-Flag or Tri-mut VCP-Flag with or without *H. pylori* infection for 24 hr. The percentage of cells in G0/G1 was examined by flow cytometry. In D–F, *p<0.05, **p<0.01 relative to cells uninfected with *H. pylori*. **§** *p*<0.01 relative to cells transfected with WT VCP-Flag. Data are expressed as means ± SD.

IPA analysis showed that VCP-binding proteins identified in *H. pylori*-infected AGS cells are involved in cell cycle progression, death of cancer cells, biosynthesis of protein, and tumorigenesis. To investigate a potential role for VCP in suppressing *H. pylori*-induced apoptosis, we knocked down Akt or VCP expression in AGS cells using the corresponding shRNA. As shown in Figure 4A, knockdown by Akt shRNA reduced Akt levels by 70% and that using VCP shRNA reduced VCP levels by 90%. Both types of knockdown cell showed a marked increase in apoptosis after *H. pylori* infection, as shown by annexin V staining and flow cytometry (Figure 4B), suggesting that VCP phosphorylation promoted cell survival. We next examined the effect of inhibition of VCP phosphorylation on *H. pylori*-stimulated apoptosis of AGS cells. *H. pylori* were co-cultured with AGS cells in the presence or absence of LY294002, then VCP phosphorylation and apoptosis were analyzed. As expected, *H. pylori*-induced VCP phosphorylation was inhibited by LY294002 (Figure S2A) and the increase in apoptosis caused by *H. pylori* infection was markedly increased by blocking of VCP phosphorylation (Figure 4C). Similarly, transfection with the Tri-mut VCP-Flag plasmid resulted in increased *H. pylori*-stimulated apoptotic activity (Figure 4D), inhibition of cell proliferation (Figure 4E), and increased G0/G1 cell cycle arrest (Figure 4F) compared to transfection with the wild-type VCP-Flag, confirming the functional involvement of the detected phosphorylation sites. Taken together, these results demonstrate that VCP expression and phosphorylation enhance the survival of AGS cells infected by *H. pylori*.

### *H. pylori* promotes the accumulation of ubiquitinated proteins in the aggresome through VCP phosphorylation

**Figure 5:**
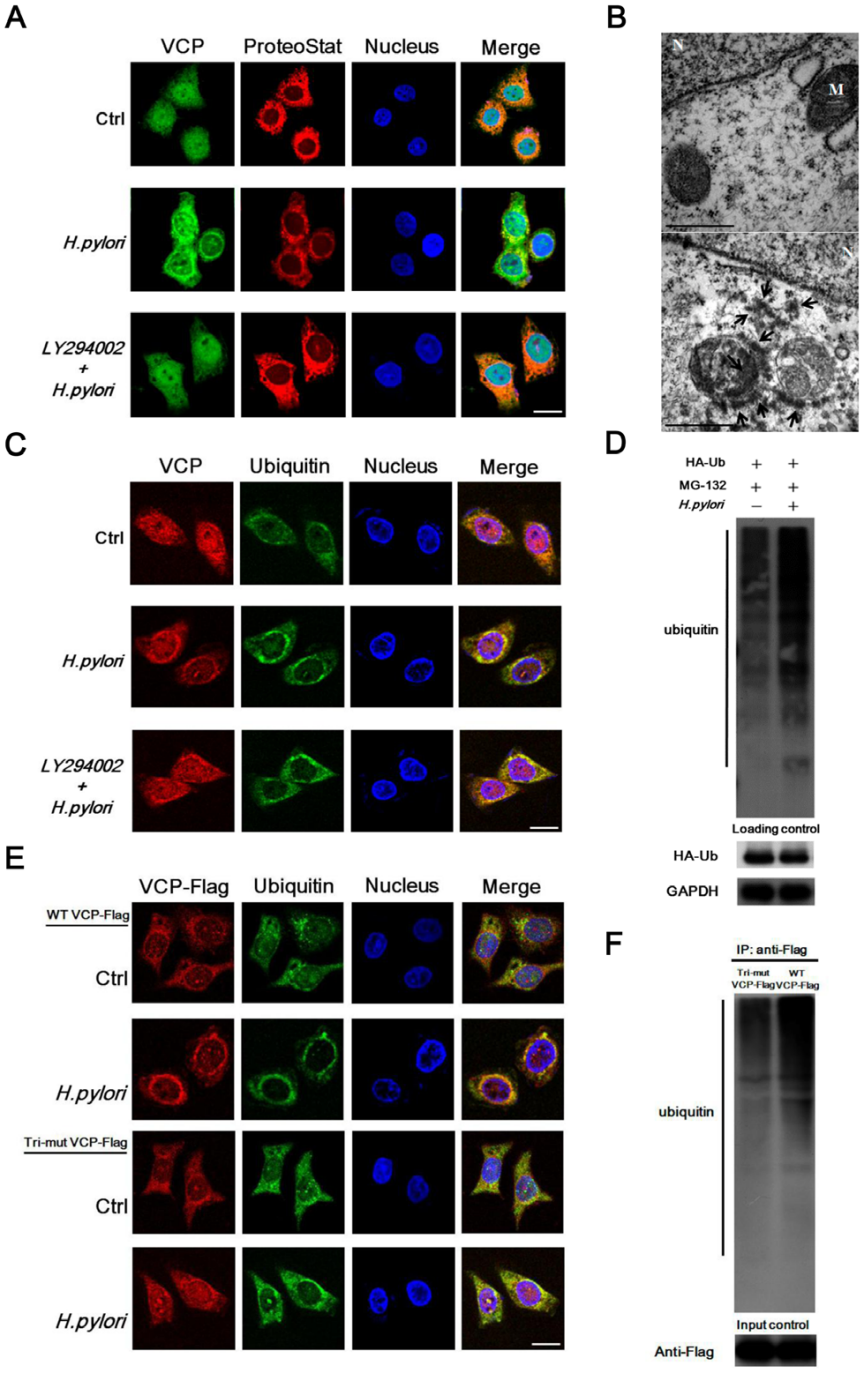
*H. pylori* induces aggresome formation through VCP phosphorylation during infection of AGS cells. *(A)* AGS cells were incubated with or without LY294002 for 3 hr, then were cultured in the presence or absence of *H. pylori* for 6 hr and double-immunostained with antibodies against VCP (green) or ProteoStat (red). *(B)* Electron micrographs of AGS cells incubated with (lower panel) or without (upper panel) *H. pylori* showing protein aggregation in the aggresome (arrows). Scale bar, 500 nm. M = Mitochondria, N = Nuclear. *(C)* AGS cells were incubated with or without LY294002 for 3 hr, then were cultured in the presence or absence of *H. pylori* for 6 hr, then were double-immunostained with antibodies against VCP (red) and ubiquitin (green). *(D)* Analysis of protein ubiquitination in AGS cells incubated with or without *H. pylori* for 6 hr. Proteasomal degradation was inhibited in both samples using 10 µM MG-132 and the cell lysates were examined by immunoblotting using anti-HA antibody. *(E)* VCP-Flag or Tri-mut VCP-Flag transfected AGS cells were incubated with or without *H. pylori* for 6 hr and then double-immunostained with antibodies against VCA (red) and ubiquitin (green). Nuclei were visualized by counterstaining with DAPI (blue), and merged immunostained images are marked as Merge. Scale bar, 10 µm. *(F)* AGS cells were transfected with VCP-Flag or Tri-mut VCP-Flag and infected with *H. pylori* and incubated with MG-132 for 6 hr, then IP was performed using anti-Flag M2 affinity gel, followed by immunoblotting using anti-ubiquitin antibody.

VCP is known to bind ubiquitinated proteins and promote their degradation in proteasomes [33]–[35]. Cells treated with proteasome inhibitors accumulate polyubiquitinated proteins and ubiquitin proteasome system (UPS) components around the centrosome in a structure termed the aggresome. To determine whether the polyubiquitinated proteins that accumulate formed an aggresome in *H. pylori*-infected AGS cells, we used immunofluorescence confocal microscopy using anti-VCP antibodies, ProteoStat, which detects aggresomes, FK2 antibody, which detects mono- and polyubiquitinated proteins, and DAPI, which detects the nucleus. As shown in Figures 5A and 5C, in control cells, VCP proteins were diffusely distributed in the cytoplasm and nucleus, but polyubiquitinated proteins were only distributed in the cytoplasm. However, in *H. pylori*-infected AGS cells, polyubiquitinated proteins accumulated and formed an aggresome and VCP was co-localized with the aggresome around the nucleus. As shown in Figure 5B, electron micrographs of *H. pylori*-infected AGS cells showed that the aggresome contained a large number of fine granules adjacent to the nucleus and surrounding mitochondria (lower panel), which were absent in non-infected control cells (upper panel). In AGS cells treated with the PI3K inhibitor LY294002 before *H. pylori* infection, VCP did not co-localize with polyubiquitinated proteins and aggresome formation was inhibited (Figure 5A and 5C). To further examine protein polyubiquitination, AGS cells transiently transfected with HA epitope-tagged ubiquitin (HA-UB) expression plasmids were incubated with the proteasome inhibitor MG-132 with or without *H. pylori* treatment and polyubiquitinated protein levels were found to be significantly increased in *H. pylori*-infected AGS cells (Figure 5D).

We then examined wild type VCP- and Tri-mut VCP-transfected AGS cells infected with *H. pylori*. As shown in Figure 5E, the results showed appreciable co-localization of VCP and ubiquitinated proteins in VCP-transfected AGS cells infected with *H. pylori*, but not in Tri-mut VCP-Flag-transfected AGS cells. Additionally, levels of polyubiquitinated proteins were reduced in Tri-mut VCP-Flag-transfected AGS cells infected with *H. pylori* (Figure 5F). These results show that *H. pylori* is a potent aggresome inducer and promotes accumulation of VCP in the aggresome through VCP phosphorylation.

### *H. pylori* stimulates degradation of cellular regulators via efficient protein ubiquitination and aggresome formation mediated by VCP phosphorylation

**Figure 6:**
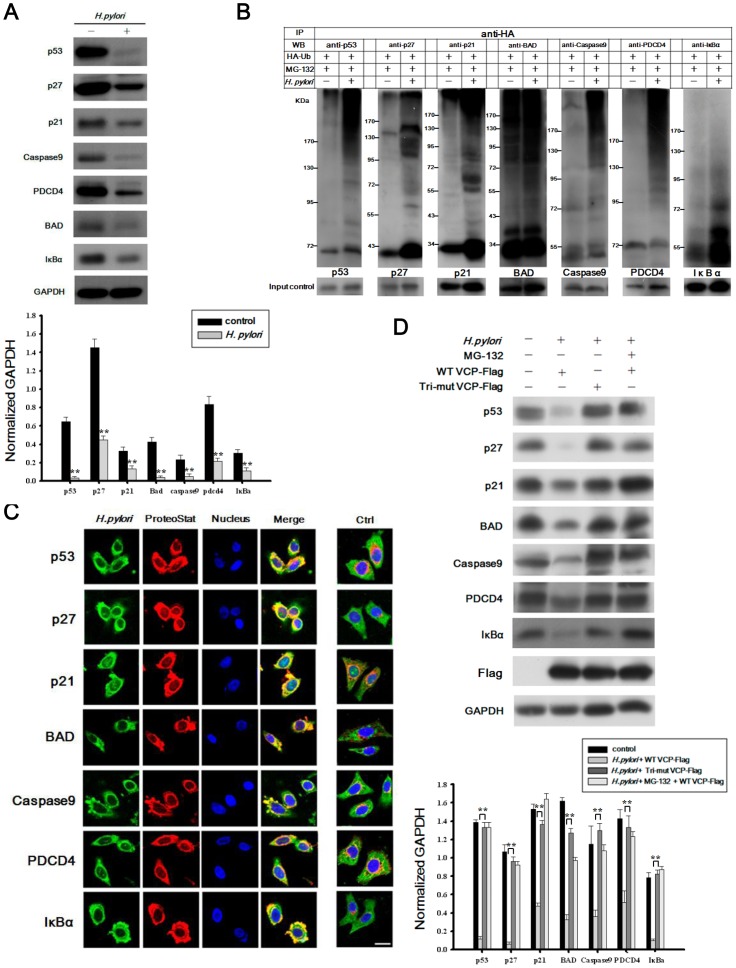
*H. pylori* induces VCP phosphorylation-mediated degradation of cellular regulators. *(A)* Immunoblotting analysis showing the expression of VCP-downstream regulatory factors in AGS cells with or without infection with *H. pylori*. The bottom panel shows densitometric results for the respective blots normalized to the GAPDH levels expressed relative to levels in the non-infected control. **p<0.01 relative to control. Data are expressed as means ± SD. *(B)* Immunoblotting analysis showing the ubiquitination of VCP-downstream regulatory factors in *H. pylori*-infected AGS cells. *(C)* AGS cells were incubated with or without *H. pylori* for 6 hr, then were double-immunostained with antibodies against cellular regulators (green) and ProteoStat (red). Nuclei were visualized by counterstaining with DAPI (blue) and the merged images are marked as Merge. *(D)* AGS cells expressing WT VCP-Flag, Tri-mut VCP-Flag or empty vector were treated with 10 µM MG-132 for 3 hr before being incubated with or without *H. pylori* for 24 hr, then were analyzed by immunoblotting. GAPDH served as the loading control. The bottom panel shows densitometric results for the respective blots normalized to the GAPDH levels and expressed relative the level in the mock control. **p<0.01 relative to cells transfected with WT VCP-Flag. Data are expressed as means ± SD.

We further examined protein levels of the cellular regulators p53, p21^Cip1^, p27^kip1^, Caspase9, PDCD4, BAD, and IκBα. As shown in Figure 6A, when AGS cells were infected with *H. pylori*, levels of p53, p21^Cip1^, p27^kip1^, Caspase9, PDCD4, BAD, and IκBα were markedly decreased compared to those in non-infected cells. Because protein ubiquitination is crucial for protein degradation, we next assessed whether *H. pylori* stimulated polyubiquitination of these proteins. As shown in Figure 6B, in AGS cells expressing HA-Ub treated with the MG-132, levels of ubiquitinated proteins were significantly increased in *H. pylori*-infected AGS cells compared to non-infected cells. Next, we examined the subcellular localization of these cellular regulators by confocal microscopy. As shown in Figure 6C, in *H. pylori*-infected AGS cells, but not in the controls, these cellular regulators were found in a perinuclear structure that colocalized with ubiquitinated proteins (aggresome). Finally, to examine the role of VCP in regulating the degradation of these cellular regulators in *H. pylori*-infected AGS cells, cells were transfected with wild type VCP-Flag or Tri-mut VCP-Flag or were treated with MG-132. As shown in Figure 6D, protein degradation was inhibited to a similar degree in cells overexpressing Tri-mut VCP-Flag and in those treated with MG-132. Together, these results provide strong support for the notion that VCP phosphorylation is involved in the degradation of p53, p21^Cip1^, p27^kip1^, Caspase9, PDCD4, BAD, and IκBα and promotes an anti-apoptotic response in *H. pylori*-infected gastric epithelial cells.

## Discussion

**Figure 7:**
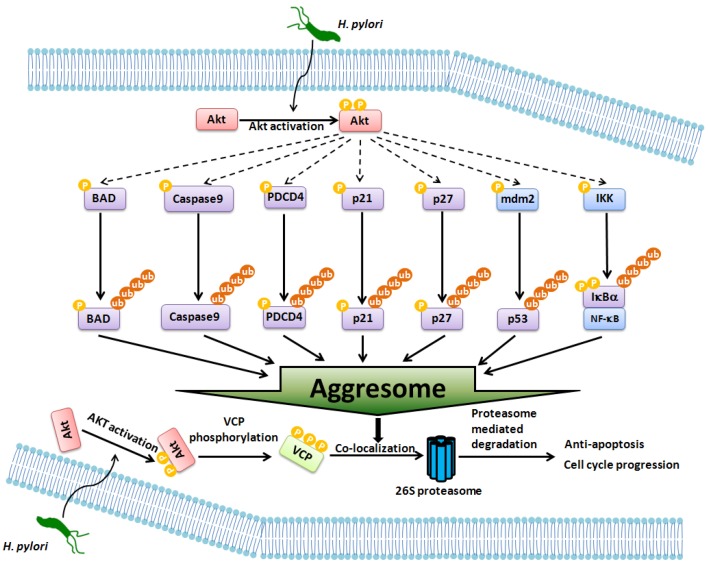
Schematic model of the hypothetical mechanism for VCP anti-apoptotic pathways in gastric epithelial cells infected with *H. pylori*. The solid arrows indicate pathways that have been previously demonstrated, while the dashed arrows indicate hypothetical links.

In this study, we demonstrated that VCP activation is a key factor promoting overall survival in gastric epithelial cells infected with *H. pylori*. Our data showed that, in AGS cells, *H. pylori* induced VCP phosphorylation as a result of activation of Akt. Activation of Akt also promoted the *H. pylori*-induced ubiquitination of the cellular regulator*s* p53, p21^Cip1^, p27^kip1^, Caspase9, PDCD4, BAD, and IκBα and the accumulation of ubiquitinated aggregates. Furthermore, we observed that phosphorylated VCP co-localized with the aggresome in the perinuclear region in *H. pylori*-infected AGS cells and triggered cellular regulator degradation, which in turn, stimulated activation of the anti-apoptotic pathway and cell cycle progression (Figure 7). These novel findings suggest that activation of VCP may be a previously unrecognized “molecular switch” determining the survival response in *H. pylori*-infected gastric epithelial cells.

Previously, Madeo F et al found Cdc48 from yeast and its mammalian homologue VCP mainly located at the endoplasmic reticulum in these cells. As soon as the cells enter mitosis, the distribution of the VCP signal changes rapidly. VCP tyrosine phosphorylation is an induction of nuclear transport and associated with relocalization to the centrosome during mitosis. [36]. In the present study, we found VCP proteins were diffusely distributed in the cytoplasm and nucleus. When *H. pylori* infected AGS cells, VCP serine phosphorylation is induced and promotes accumulation of VCP in the aggresome around the nucleus. [37]–[39]. Reduction of VCP phosphorylation levels, aggresome formation was inhibited. These data indicate that VCP participates cellular function depends on its intracellular localization, which in turn is regulated by different phosphorylation.

A bioinformatic analysis of the VCP-interacting proteins identified in the present study showed that many were involved in the PI3K/Akt signaling canonical pathway. The serine/threonine kinase Akt is an important mediator of cell survival and cell proliferation [32], [40] and VCP has been shown to be a target of Akt signaling in neuronal cells and breast cancer cells [32], [40], [41]. In our study, *H. pylori* infection led to increased Akt kinase activity and increased VCP phosphorylation, and these effects were decreased by inhibition of the PI3K/Akt pathway using LY294002. Our data also showed that these three serine residues are crucial for VCP regulation by Akt. Additionally, we found that VCP phosphorylation, mediated by Akt, induced perinuclear aggresome formation. Ubiquitinated proteins that accumulate in cells as a consequence of impaired UPS function usually join to form perinuclear aggresomes [42], [43]. The accumulation of ubiquitinated proteins in perinuclear aggresome was co-localized with VCP in *H. pylori*-infected AGS cells. VCP phosphorylation may result in its binding ubiquitinated proteins and facilitating their delivery to the aggresome following *H. pylori* stimulation. This suggests that phospho-VCP may be involved in protein aggregate formation or aggregate clearance from the cell. We further demonstrated that knockdown of VCP expression or inhibition of its phosphorylation reduced the proliferation of AGS cells and induced their apoptosis. Our data suggest that VCP regulates a number of critical cellular processes involved in survival and proliferation. VCP participates in the delivery of degradation-fated ubiquitinated proteins to the 26S proteasome [13]. *H. pylori* infection-induced aggresome formation may also contribute to these downstream events. Thus, increased phosphorylation of VCP, mediated by Akt, promotes cell proliferation by switching the cell towards the survival pathway.

The NF-κB signaling pathway is the main signaling pathway activated in *H.pylori*-infected AGS cells [44]. In our study, we showed that *H. pylori* activated NF-κB via a signaling pathway involving phosphorylation of Akt and IκBα ubiquitination. Furthermore, we found that VCP phosphorylation played a key role in IκBα degradation. These data indicate that NF-κB activation and its effects on cell survival are functionally regulated through PI3K/AKT-dependent VCP phosphorylation in *H. pylori*-infected cells.

Protein ubiquitination and degradation play a pivotal role in numerous biological functions [45]–[47]. Protein ubiquitination was originally thought to target proteins for 26S proteasome-dependent degradation [48]. Akt-mediated phosphorylation of a number of targets causes their degradation by ubiquitination, and this regulation is associated with the distribution of prosurvival and antiapoptotic signals [49]. In our study, multiple VCP-interacting proteins involved in the Akt pathway were identified in *H. pylori*-infected cells. BAD, a member of the BCL2 family, is regulated by Akt phosphorylation, which inactivates its proapoptotic function [50], [51]. Programmed cell death 4 (PDCD 4) is a tumor suppressor protein that was originally identified as a neoplastic transformation inhibitor [52]. A previous study demonstrated that degradation of PDCD 4 in mitogen-stimulated cells is required for efficient protein translation and thus essential for cell growth or proliferation [52]. PKC-mediated activation of the PI3K/Akt signaling pathway leads to phosphorylation of PDCD4, thereby targeting it for proteasomal degradation [52]–[54]. Caspase9, the initiator caspase of the mitochondrial apoptotic pathway, is one of the substrates for XIAP E3 ubiquitin ligase, which promotes polyubiquitination of Caspase 9 via the PI3K/Akt pathway [55], [56].

Phosphorylation of cytoplasmic p21^Cip1^ is mediated by activation of Akt. Akt directly phosphorylates p21^Cip1^, resulting in its ubiquitin-dependent proteolysis and in cell cycle progression [57]. Akt activation also induces phosphorylation of p27^kip1^, which then moves to the cytoplasm and is ubiquitinated and degraded, again resulting in cell cycle progression [57]. Recent finding showed that the CagA+ *H. pylori* strain was able to induce an significant transient elevation of Akt phosphorylation in the infected AGS cells, but the Cag A− *H. pylori* strain was failed. Furthermore, they also found that the p21^Cip1^ and p27^kip1^ mRNA levels in CagA+ *H. pylori* infected AGS cells declined, while the p21^Cip1^ mRNA level in CagA− *H. pylori* infected AGS cells increased [58]. Moreover, CagA+ *H. pylori* infection activates Akt1, resulting in phosphorylation and activation E3 ubiquitin ligase MDM2 and subsequent degradation of p53 in gastric epithelial cells [59]. Taken together, CagA might be an important factor on the elevation of Akt phosphorylation level. The stability of p21^Cip1^, p27^kip1^, and p53 is tightly and differentially regulated by the ubiquitin proteasome system in a manner that depends on VCP phosphorylation activated by CagA+ *H. pylori* infection [31], [60], [61].

Our results demonstrated that ubiquitination and degradation of BAD, PDCD4, Caspase9, p21^Cip1^, p27^kip1^, and p53 is crucial for activation of the PI3K/Akt pathway in *H. pylori*-infected AGS cells, which contributes to cell survival and cell cycle progression. Supporting this, we found that phospho-VCP interacted with these proteins in vitro and enhanced their degradation, whereas overexpression of Tri-mut VCP could not be phosphorylated by Akt and failed to bind ubiquitinated proteins, then inhibiting their degradation. These results suggest that VCP regulates many cellular responses that are associated with gastric cancer development. Further studies are needed to determine whether the Akt-enhanced ubiquitination of these proteins also contributes to *H. pylori*-induced cellular responses.

In conclusion, we propose a novel mechanism linking VCP phosphorylation and the degradation of ubiquitinated aggregates. Intracellular ubiquitination of cellular regulators, such as p53, p21^Cip1^, p27^kip1^, Caspase9, PDCD4, BAD, and IκBα, which associate directly with phospho-VCP, leads to their translocation to the perinuclear region in association with phospho-VCP. The perinuclear translocation of VCP results in the degradation of accumulated aggregates by the ubiquitin/proteasome degradation pathway. The cross-regulation between phosphorylation and ubiquitination appears to be a key mechanism generating an anti-apoptotic effect and promoting cancer cell survival and the proteins in this signaling pathway should be considered as potential targets for gastric cancer therapy.

## Supporting Information

Figure S1 **Co-regulation analysis of cell survival in** ***H. pylori*** **-infected AGS cells.** The Akt signaling pathways were generated using Ingenuity pathway analysis. Proteins selected as cellular regulators included p53, p21^Cip1^, p27^kip1^, Caspase9, PDCD4, BAD, and IκBα.
(TIF)

Figure S2 **Analysis of VCP phosphorylation sites in** ***H. pylori*** **-infected AGS cells.** *(A)* AGS cells overexpressing VCP-Flag were left untreated or were pretreated with 10 µM LY294002 for 3 hr prior to incubation for 6 hr with or without *H. pylori*., then IP was performed using anti-Flag M2 affinity gel, followed by immunoblotting using anti-Flag, anti-pAkt, and anti-PAS antibodies. 1% of input (right) was subjected to immunoblotting. *(B)* Putative Akt kinase phosphorylation consensus logos in VCP identified using Scansite software; the three identified possible sites are AATNRPNS**352**, AMRFARRS**746**, and RFARRSVS**748**. *(C)* AGS cells were transfected with vector encoding wild type VCP (WT VCP-Flag), the single mutants VCPs VCP-Flag^S352A^, VCP-Flag^S746A^, and VCP-Flag^S748A^, the double mutants VCPs VCP-Flag^S352AS746A^, VCP-Flag^S352AS748A^, and VCP-Flag^S746AS748A^, or the triple mutant VCP VCP-Flag^S352AS746AS748^A, then were incubated with or without *H. pylori* for 6 hr, when IP was performed using anti-Flag M2 affinity gel, followed by immunoblotting using anti-Flag and anti-PAS antibodies.
(TIF)

Materials and Methods S1 (DOCX)

Table S1 **Primer sequences for VCP and site-specific VCP mutant constructs**
(PDF)

Table S2 **List of 288 VCP interaction proteins immunoprecipitated with anti-Flag M2 affinity gel from H. pylori infected AGS cells.**
(PDF)

Table S3 **Top high-level functions identified by Ingenuity global function analysis of VCP-interacting proteins in** ***H. pylori*** **-infected AGS cells.**
(PDF)
