# Supplementary figures and images for: VCP Phosphorylation-Dependent Interaction Partners Prevent Apoptosis in Helicobacter pylori-Infected Gastric Epithelial Cells
Source: PLoS One. 2013 Jan 31;8(1):e55724. doi: 10.1371/journal.pone.0055724 (PMC3561343; doi:10.1371/journal.pone.0055724)

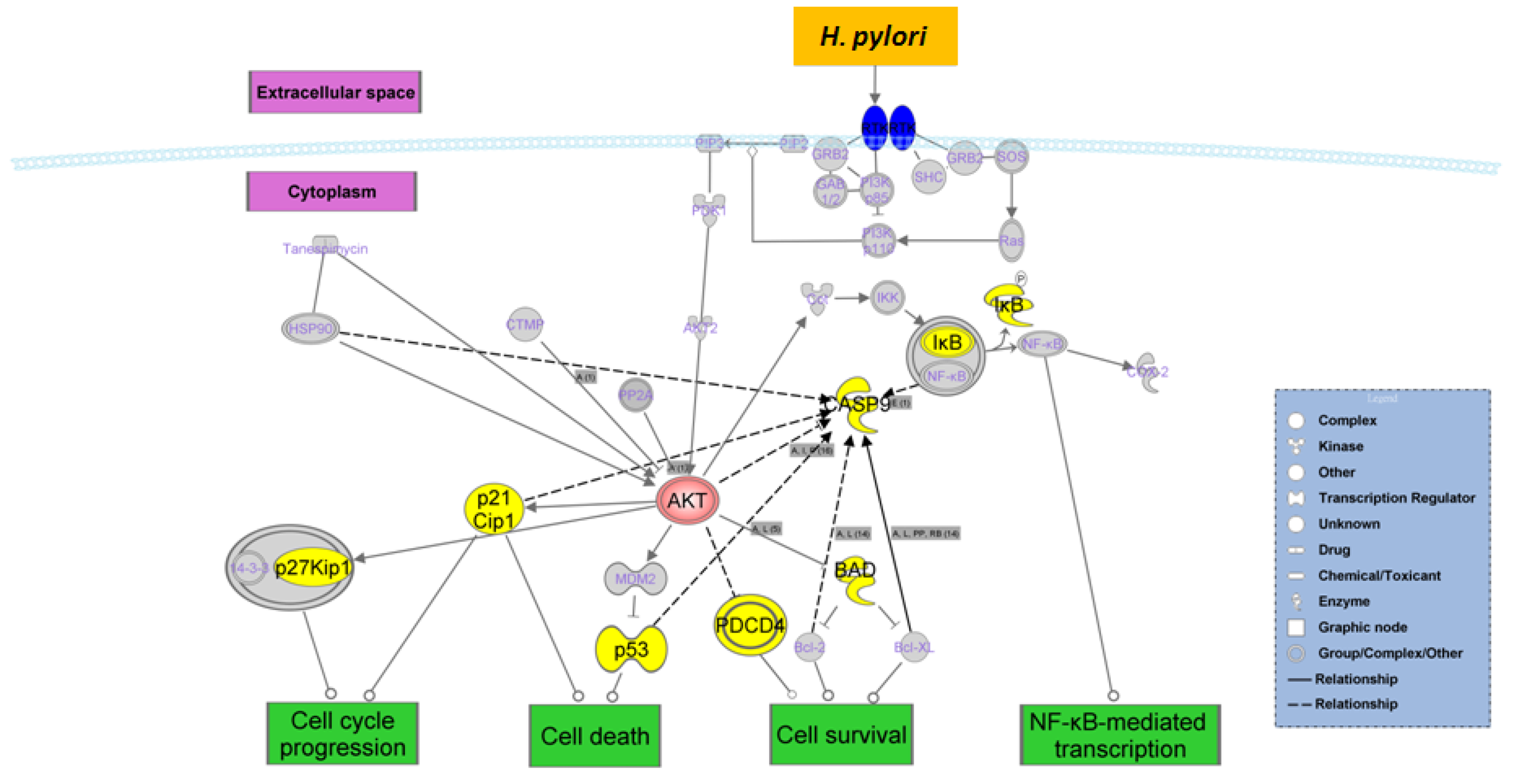

Supplement: Figure S1 — Co-regulation analysis of cell survival in H. pylori -infected AGS cells. The Akt signaling pathways were generated using Ingenuity pathway analysis. Proteins selected as cellular regulators included p53, p21Cip1, p27kip1, Caspase9, PDCD4, BAD, and IκBα. (TIF) [file pone.0055724.s001.tif]

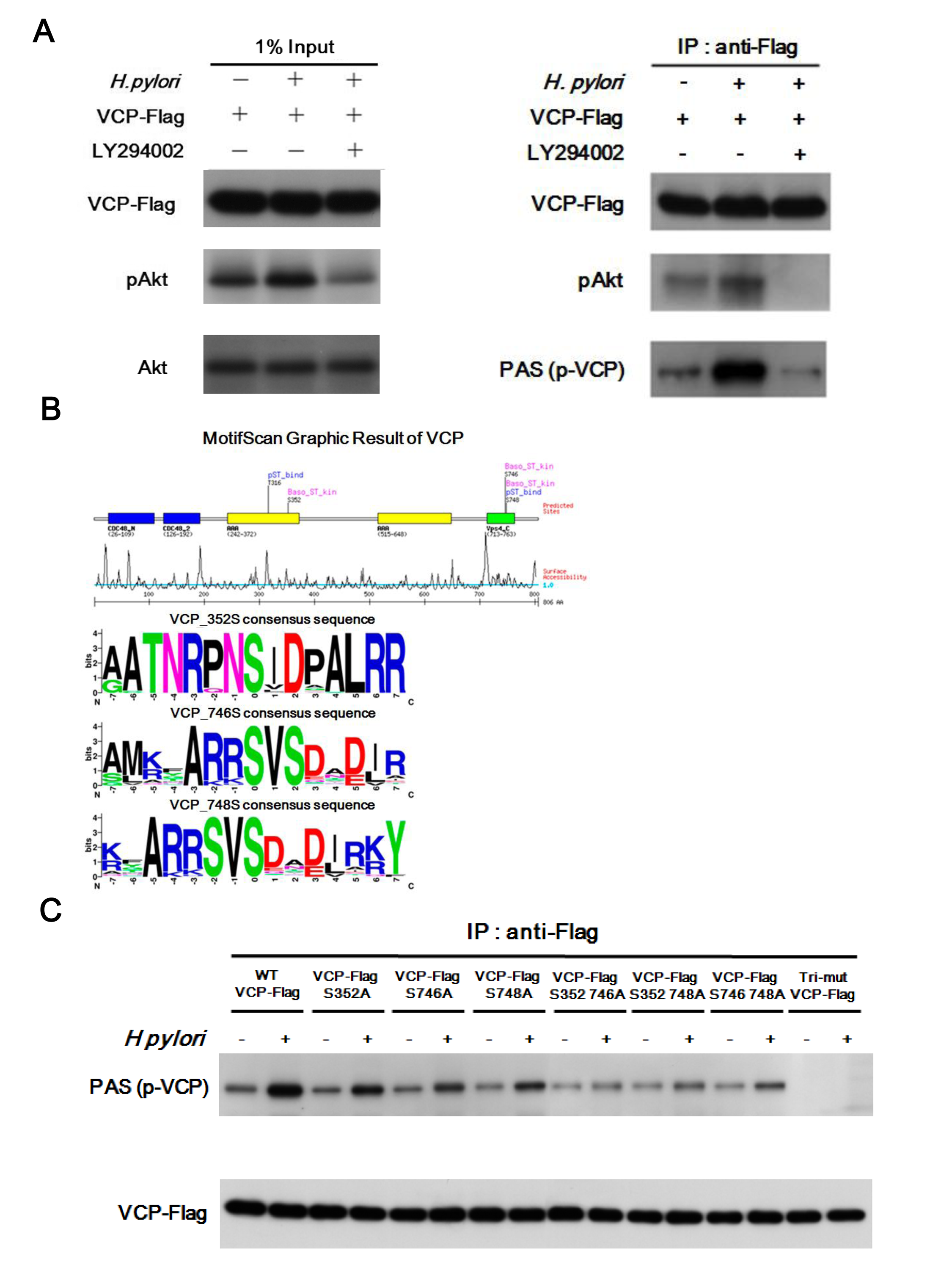

Supplement: Figure S2 — Analysis of VCP phosphorylation sites in H. pylori -infected AGS cells. (A) AGS cells overexpressing VCP-Flag were left untreated or were pretreated with 10 µM LY294002 for 3 hr prior to incubation for 6 hr with or without H. pylori., then IP was performed using anti-Flag M2 affinity gel, followed by immunoblotting using anti-Flag, anti-pAkt, and anti-PAS antibodies. 1% of input (right) was subjected to immunoblotting. (B) Putative Akt kinase phosphorylation consensus logos in VCP identified using Scansite software; the three identified possible sites are AATNRPNS352, AMRFARRS746, and RFARRSVS748. (C) AGS cells were transfected with vector encoding wild type VCP (WT VCP-Flag), the single mutants VCPs VCP-FlagS352A, VCP-FlagS746A, and VCP-FlagS748A, the double mutants VCPs VCP-FlagS352AS746A, VCP-FlagS352AS748A, and VCP-FlagS746AS748A, or the triple mutant VCP VCP-FlagS352AS746AS748A, then were incubated with or without H. pylori for 6 hr, when IP was performed using anti-Flag M2 affinity gel, followed by immunoblotting using anti-Flag and anti-PAS antibodies. (TIF) [file pone.0055724.s002.tif]
