# Supplementary material for: VCP Phosphorylation-Dependent Interaction Partners Prevent Apoptosis in Helicobacter pylori-Infected Gastric Epithelial Cells
Source: PLoS One. 2013 Jan 31;8(1):e55724. doi: 10.1371/journal.pone.0055724 (PMC3561343; doi:10.1371/journal.pone.0055724)
Supplement: Materials and Methods S1 — (DOCX) [file pone.0055724.s003.docx]

**Supplemental Materials and Methods**

***Inhibitors, Antibodies, and Reagents* -** Protease and phosphatase inhibitor cocktails, LY294002 (a phosphatidylinositol 3-kinase (PI3K) inhibitor), and MG-132 (a 26S proteasome inhibitor) were obtained from Sigma-Aldrich (St. Louis, MO). Polyclonal rabbit anti-phospho-Akt-substrate (PAS) and anti-Akt antibodies were from Cell Signaling Technology (Beverly, MA). Polyclonal rabbit anti-p53, anti-p27, anti-p21, anti-PDCD4, anti-BAD, and anti-IκBα antibodies were from GeneTex (Irvine, CA). The anti*-*Flag M2 affinity gel and horseradish peroxidase (HRP)-conjugated mouse anti-Flag M2 antibodies were from Sigma-Aldrich. Polyclonal rabbit anti-VCP, anti-pAkt1 (Ser473), anti-HA (Y-11), and anti-GAPDH antibodies and mouse monoclonal anti-HA (F-7) antibody were from Santa Cruz (Santa Cruz, CA). Mouse monoclonal anti-ubiquitin antibody, TRITC -conjugated anti-rabbit or anti-mouse IgG and FITC-conjugated anti-rabbit or anti-mouse IgG antibodies were from Millipore (Bedford, MA). HRP-conjugated anti-mouse IgG or anti-rabbit IgG antibodies were from Jackson Immunoresearch Laboratories Inc. (West Grove, PA), The enhanced chemiluminescence (ECL) kits were from Amersham Biosciences and the BCA^TM^ protein assay kits from Pierce (Rockford, IL). Lipofectamine 2000 was from Invitrogen (Carlsbad, CA). The Phusion® site-directed mutagenesis kits were from New England Biolabs, Inc. (Vantaa, Finland). The PVDF membranes were from Millipore (Bedford, MA). Aggresomes were detected using ProteoStat kits from Enzo Life Sciences, PA. Nuclei were detected using DAPI (Invitrogen*,* Inc.). Annexin V-FITC and propidium iodide were from Calbiochem/EMD Biosciences (Darmstadt, Germany) and the dual-luciferase reporter assay system and MTS cell proliferation assay kits were from Promega Corp (Madison, WI).

***Clones and Constructs -*** A 2.4 kb *Hind*III-*BamH*I fragment containing the full-length human *VCP* cDNA was generated by PCR using the forward and reverse primers shown in Supplemental Table S1 and a human AGS gastric adenocarcinoma cell cDNA library as template. The amplified cDNA fragment was subcloned into the mammalian expression vector pCMV14-3xFlag (Sigma-Aldrich) to generate plasmid pCMV14-VCP-3xFlag, which was then digested with *Hind*III and *EcoRV* to release the VCP-Flag coding region, which was inserted into the AdTrack.CMV plasmid (Stratagene, La Jolla, CA) to form plasmid AdTrack-CMV-VCP-3xFlag which was then used, together with the homologous recombinant adenovirus genomic plasmid pAdEasy (Stratagene, La Jolla, CA), to co-transfect BJ5183 bacteria. After recombination with the wild type adenovirus DNA, the viruses were propagated, purified, and titrated.

The site-specific mutants of VCP used were the single mutants S352A, S746A, and S748A, the double mutants S352A/S746A, S352A/S748A, and S746A/S748A, and the triple mutant S352A/S746A/S748A and were generated using Phusion DNA Polymerase and a Phusion® Site-Directed mutagenesis kit with pCMV14-VCP-3xFlag as the template. cDNAs coding for the mutants were generated using the primers shown in Table S1 and a human AGS gastric adenocarcinoma cell cDNA library as template. The PCR product was inserted in the p3xFlag-CMV14 vector and amplified in *Escherichia coli*. Cloning and mutation were confirmed by DNA sequencing. pCMV-VCP-3xFlag, pCMV-VCP-3xFlag^S352A^, pCMV-VCP-3xFlag^S746A^, pCMV-VCP-3xFlag^S748A^, pCMV-VCP-3xFlag^S352A/S746A^, pCMV-VCP-3xFlag^S352A/S748A^, pCMV-VCP-3xFlag^S746A/S748A^ and pCMV-VCP-3xFlag^S352A/S746A/S748A^ were then expressed in AGS cells.

Lentivirus vectors expressing short hairpin RNA (shRNA) targeting AKT (target sequence, 5’-GGA CAA GGA CGG GCA CAT TAA-3’) or VCP (target sequence, 5’-CCT ATC AAC AGC CAT TCT CAA-3’) were provided by the RNAi core facility in the Genomics Research Center, Academia Sinica.
