# Supplementary material for: VCP Phosphorylation-Dependent Interaction Partners Prevent Apoptosis in Helicobacter pylori-Infected Gastric Epithelial Cells
Source: PLoS One. 2013 Jan 31;8(1):e55724. doi: 10.1371/journal.pone.0055724 (PMC3561343; doi:10.1371/journal.pone.0055724)
Supplement: Table S1 — Primer sequences for VCP and site-specific VCP mutant constructs (PDF) [file pone.0055724.s004.pdf]

Table S1. Primer sequences for VCP and site-specific VCP mutant constructs

Gene	Direction	Sequence (5′→3′)
Human *VCP*	Forward	AAGCTTATGGCTTCTGGAGCCGATTCAAAAGG-3’
	Reverse	GGATCCGCCATACAGGTCATCATCATTGTCTTC-3’
Human *VCP* ^{S352A}	Forward	AACAGACCCAACGCCATTGACCCA
	Reverse	GGTTGCTGCCATAACAATCACATG
Human *VCP* ^{S746A}	Forward	GCGCGCCGTGCTGTCAGTGACAAT
	Reverse	AAAGCGCATGGCTTCTTCAAAGTG
Human *VCP* ^{S748A}	Forward	CGTTCTGTCAGCGACAATGACATT
	Reverse	GCGCGCAAAGCGCATGGCTTCTTC
Human *VCP* $^{S746A/S748A}$	Forward	GCGCGCCGTGCTGTCAGCGACAAT
	Reverse	AAAGCGCATGGCTTCTTCAAAGTG
