# Supplementary material for: VCP Phosphorylation-Dependent Interaction Partners Prevent Apoptosis in Helicobacter pylori-Infected Gastric Epithelial Cells
Source: PLoS One. 2013 Jan 31;8(1):e55724. doi: 10.1371/journal.pone.0055724 (PMC3561343; doi:10.1371/journal.pone.0055724)
Supplement: Table S2 — List of 288 VCP interaction proteins immunoprecipitated with anti-Flag M2 affinity gel from H. pylori infected AGS cells. (PDF) [file pone.0055724.s005.pdf]

Supplemental Table 2. List of 288 VCP interaction proteins immunoprecipitated with anti-Flag M2 affinity gel from *H. pylori* infected AGS cells.

Accession Number	# AAs	MW [kDa]	calc. pI	gene name	Description	[a]Coverage	[b]PSMs	[c]Peptides	[d]Score
P61604	102	10.9	8.9	HSPE1	10 kDa heat shock protein, mitochondrial	13.73	2	1	66.5
P31946	246	28.1	4.8	YWHAB	14-3-3 protein beta/alpha	20.73	4	1	59.2
P62258	255	29.2	4.7	YWHAE	14-3-3 protein epsilon	18.04	6	3	150.5
Q04917	246	28.2	4.8	YWHAH	14-3-3 protein eta	20.73	5	3	121.5
P61981	247	28.3	4.9	YWHAG	14-3-3 protein gamma	13.36	4	1	91.2
P27348	245	27.7	4.8	YWHAQ	14-3-3 protein theta	31.02	8	5	153.6
P63104	245	27.7	4.8	YWHAZ	14-3-3 protein zeta/delta	20.82	6	3	161.7
Q9C0C2	1729	181.7	4.9	TNKS1BP1	182 kDa tankyrase-1-binding protein	4.05	7	4	195.5
P62277	151	17.2	10.5	RPS13	40S ribosomal protein S13	8.61	2	2	49.3
P46781	194	22.6	10.7	RPS9		17.53	5	5	105.0
P08195	630	68.0	5.0	SLC3A2	4F2 cell-surface antigen heavy chain	2.22	2	1	54.4
P54619	331	37.6	6.9	PRKAG1	5'-AMP-activated protein kinase subunit gamma-1	8.76	2	2	66.7
Q96D46	503	57.6	7.1	NMD3	60S ribosomal export protein NMD3	3.38	2	1	60.0
F8W181	226	25.9	10.7	RPL6	60S ribosomal protein L6	11.06	3	2	32.5
P11021	654	72.3	5.2	HSPA5	78 kDa glucose-regulated protein	18.2	11	6	202.6
Q13085	2346	265.4	6.4	ACACA	Acetyl-CoA carboxylase 1	1.79	4	3	81.0
P60709	375	41.7	5.5	ACTB	Actin, cytoplasmic 1	38.4	17	7	266.1
O15144	300	34.3	7.4	ARPC2	Actin-related protein 2/3 complex subunit 2	6.67	4	2	41.2
P62330	175	20.1	9.0	ARF6	ADP-ribosylation factor 6	24	3	3	68.8
Q8N6H7	521	56.7	8.0	ARFGAP2	ADP-ribosylation factor GTPase-activating protein 2	9.4	5	3	87.5
Q9UJY5	639	70.3	5.3	GGA1	ADP-ribosylation factor-binding protein GGA1	12.05	11	6	172.0
Q9UJY4	613	67.1	6.6	GGA2	ADP-ribosylation factor-binding protein GGA2	11.42	7	5	121.2
P12814	892	103.0	5.4	ACTN1	Alpha-actinin-1	1.12	1	1	81.4
P35611	737	80.9	5.8	ADD1	Alpha-adducin	9.23	5	4	74.5
P54920	295	33.2	5.4	NAPA	Alpha-soluble NSF attachment protein	3.73	2	1	43.1
Q96Q42	1657	183.5	6.3	ALS2	Alsin	0.66	1	1	42.9
Q9UJX3	599	66.8	5.6	ANAPC7	Anaphase-promoting complex subunit 7	7.18	7	3	106.8
Q8IWZ3	2542	269.3	5.7	ANKHD1	Ankyrin repeat and KH domain-containing protein 1	0.51	1	1	42.3
H0YMM1	149	16.4	5.9	ANXA2	Annexin	14.09	6	2	6.8
P08758	320	35.9	5.0	ANXA5	Annexin A5	17.19	5	4	102.6
P20073	488	52.7	5.7	ANXA7	Annexin A7	10.25	6	4	134.9
Q10567	949	104.6	5.1	AP1B1	AP-1 complex subunit beta-1	12.54	19	3	347.8
O94973	939	103.9	7.0	AP2A2	AP-2 complex subunit alpha-2	5.32	5	3	83.8
Q07812	192	21.2	5.2	BAX	Apoptosis regulator BAX	17.19	4	2	106.7
Q9UBB4	475	53.5	5.2	ATXN10	Ataxin-10	23.16	15	8	295.7
P54252	364	41.8	4.9	ATXN3	Ataxin-3	3.3	2	1	51.3
Q96S55	665	72.1	6.1	WRNIP1	ATPase WRNIP1	10.98	12	5	266.8
O95816	211	23.8	6.7	BAG2	BAG family molecular chaperone regulator 2	18.01	3	2	65.4
Q9Y6E2	419	48.1	6.7	BZW2	Basic leucine zipper and W2 domain-containing protein 2	7.16	2	1	39.3
P35613	385	42.2	5.7	BSG	Basigin	7.27	2	2	40.1
Q9NYF8	920	106.1	10.0	BCLAF1	Bcl-2-associated transcription factor 1	18.37	25	12	304.1
Q8WYA6	563	65.1	5.0	CTNNBL1	Beta-catenin-like protein 1	5.15	4	3	82.7
P55957	195	22.0	5.4	BID	BH3-interacting domain death agonist	22.05	5	3	125.3

Q9UHR4	511	56.8	8.7	BAIAP2L1	Brain-specific angiogenesis inhibitor 1-associated protein 2-like protein 1	10.37	5	3	113.0
Q6PJG6	821	88.1	5.3	BRAT1	BRCA1-associated ATM activator 1	5.12	4	3	98.2
Q9P287	314	36.0	4.6	BCCIP	BRCA2 and CDKN1A-interacting protein	7.32	1	1	63.8
Q9Y6D5	1785	201.9	6.3	ARFGEF2	Brefeldin A-inhibited guanine nucleotide-exchange protein 2	2.07	3	3	80.4
Q9Y376	341	39.8	6.9	CAB39	Calcium-binding protein 39	3.23	2	1	53.0
P27824	592	67.5	4.6	CANX	Calnexin	5.74	3	3	72.8
P13861	404	45.5	5.1	PRKAR2A	cAMP-dependent protein kinase type II-alpha regulatory subunit	7.18	5	2	124.8
P67870	215	24.9	5.6	CSNK2B	Casein kinase II subunit beta	19.53	3	3	47.6
Q9H8G2	361	38.3	4.7	CAAP1	Caspase activity and apoptosis inhibitor 1	4.43	2	1	44.9
P55210	253	27.8	7.3	CASP7	Caspase-7	16.12	5	2	65.3
P21964	271	30.0	5.5	COMT	Catechol O-methyltransferase	29.89	6	5	115.6
P35221	906	100.0	6.3	CTNNA1	Catenin alpha-1	6.29	8	4	133.9
O60716	968	108.1	6.2	CTNND1	Catenin delta-1	2.38	3	2	64.1
Q9H9A5	744	82.3	7.8	CNOT10	CCR4-NOT transcription complex subunit 10	5.65	6	3	109.0
Q9NZN8	540	59.7	7.7	CNOT2	CCR4-NOT transcription complex subunit 2	5.37	4	2	97.1
Q9UFF9	292	33.5	4.8	CNOT8	CCR4-NOT transcription complex subunit 8	8.9	3	2	65.8
Q00587	391	40.3	7.2	CDC42EP1	Cdc42 effector protein 1	4.86	1	1	53.3
Q9H3Q1	356	38.0	5.2	CDC42EP4	Cdc42 effector protein 4	13.76	6	3	176.9
Q9NXV6	580	61.1	9.0	CDKN2AIP	CDKN2A-interacting protein	3.62	1	1	53.1
Q96AQ7-3	129	14.0	10.1	CIDEC	Cell death activator CIDE-3	9.3	1	1	3.3
Q92600	299	33.6	8.0	RQCD1	Cell differentiation protein RCD1 homolog	17.73	5	4	119.7
P30260	824	91.8	7.0	CDC27	Cell division cycle protein 27 homolog	4.98	5	3	142.9
Q9NX58	379	43.6	9.5	LYAR	Cell growth-regulating nucleolar protein	3.69	2	1	59.2
P04637	393	43.6	6.8	TP53	Cellular tumor antigen p53	27.23	9	7	213.0
Q9H3R5	247	28.5	5.3	CENPH	Centromere protein H	10.53	3	2	69.9
Q53EZ4	464	54.1	7.0	CEP55	Centrosomal protein of 55 kDa	8.19	5	3	114.0
Q8NI60	647	71.9	7.0	ADCK3	Chaperone activity of bc1 complex-like, mitochondrial	2.47	2	1	44.0
Q7Z460	1538	169.3	9.0	CLASP1	CLIP-associating protein 1	13.98	18	14	367.0
O14579	308	34.5	5.1	COPE	Coatomer subunit epsilon	37.34	7	4	206.6
Q8N3U4	1231	141.2	5.4	STAG2	Cohesin subunit SA-2	4.39	5	2	84.9
Q96JG6	964	111.1	6.2	CCDC132	Coiled-coil domain-containing protein 132	3.22	4	2	88.2
Q9H6E4	229	26.5	8.9	CCDC134	Coiled-coil domain-containing protein 134	13.54	2	2	54.6
P38432	576	62.6	9.1	COIL	Coilin	2.95	1	1	32.0
Q8N668	190	21.2	6.2	COMMD1	COMM domain-containing protein 1	18.42	3	2	74.4
Q7Z4G1	85	9.6	6.0	COMMD6	COMM domain-containing protein 6	28.24	3	2	102.8
Q15003	741	82.5	5.1	NCAPH	Condensin complex subunit 2	6.34	4	3	86.1
Q9H9E3	785	89.0	5.2	COG4	Conserved oligomeric Golgi complex subunit 4	6.62	8	4	153.4
Q9UP83	839	92.7	6.6	COG5	Conserved oligomeric Golgi complex subunit 5	7.03	6	4	97.7
Q9UNS2	423	47.8	6.7	COPS3	COP9 signalosome complex subunit 3	15.37	7	4	184.3
Q9UBW8	275	30.3	8.2	COPS7A	COP9 signalosome complex subunit 7a	5.82	2	1	51.5
Q93034	780	90.9	7.9	CUL5	Cullin-5	10.77	9	6	159.2
Q14999	1698	191.0	5.9	CUL7	Cullin-7	2.53	6	2	44.9
P06493	297	34.1	8.4	CDK1	Cyclin-dependent kinase 1	25.25	6	5	156.2
O14519	115	12.4	9.4	CDK2AP1	Cyclin-dependent kinase 2-associated protein 1	11.3	2	1	78.4
P11802	303	33.7	7.0	CDK4	Cyclin-dependent kinase 4	8.25	3	1	92.3
Q13409	638	71.4	5.2	DYNC1I2	Cytoplasmic dynein 1 intermediate chain 2	7.68	4	3	114.4

Q9Y6G9	523	56.5	6.4	DYNC1LI1	Cytoplasmic dynein 1 light intermediate chain 1	12.81	7	4	149.6
Q5BKZ1	582	65.6	5.1	ZNF326	DBIRD complex subunit ZNF326	2.06	2	1	47.5
Q9UKG1	709	79.6	5.4	APPL1	DCC-interacting protein 13-alpha	5.08	4	2	102.8
Q96N67	2140	242.4	6.8	DOCK7	Dedicator of cytokinesis protein 7	2.29	5	4	103.8
P54886	795	87.2	7.1	ALDH18A1	Delta-1-pyrroline-5-carboxylate synthase	5.66	7	4	183.5
P15924	2871	331.6	6.8	DSP	Desmoplakin	15.12	43	30	735.1
P18858	919	101.7	5.6	LIG1	DNA ligase 1	4.03	2	2	39.2
P11387	765	90.7	9.3	TOP1	DNA topoisomerase 1	2.61	2	1	43.0
P30876	1174	133.8	6.9	POLR2B	DNA-directed RNA polymerase II subunit RPB2	4.43	4	3	52.2
Q9BUI4	534	60.6	7.3	POLR3C	DNA-directed RNA polymerase III subunit RPC3	5.24	2	1	77.1
P04843	607	68.5	6.4	RPN1	Dolichyl-diphosphooligosaccharide--protein glycosyltransferase subunit 1	8.57	4	3	81.0
Q02750	393	43.4	6.6	MAP2K1	Dual specificity mitogen-activated protein kinase kinase 1	21.37	8	3	142.0
P33981	857	97.0	8.2	TTK	Dual specificity protein kinase TTK	1.98	2	1	69.5
Q99956	384	41.8	6.1	DUSP9	Dual specificity protein phosphatase 9	11.2	7	2	74.0
Q9UQ16	869	97.7	8.4	DNM3	Dynamin-3	8.29	6	1	115.0
Q14258	630	70.9	8.1	TRIM25	E3 ubiquitin/ISG15 ligase TRIM25	1.9	2	1	78.4
Q9UKV5	643	72.9	6.4	AMFR	E3 ubiquitin-protein ligase AMFR	1.4	1	1	0.0
Q9Y4X5	557	64.1	5.1	ARIH1	E3 ubiquitin-protein ligase ARIH1	2.51	2	1	69.8
O95376	493	57.8	5.6	ARIH2	E3 ubiquitin-protein ligase ARIH2	3.45	2	1	51.1
Q7Z6Z7	4374	481.6	5.2	HUWE1	E3 ubiquitin-protein ligase HUWE1	0.89	3	2	45.8
Q9P0J7	381	41.9	5.7	KCMF1	E3 ubiquitin-protein ligase KCMF1	8.14	4	2	102.5
Q00987	491	55.2	4.7	MDM2	E3 ubiquitin-protein ligase Mdm2	3.26	2	1	106.4
Q99496	336	37.6	6.8	RNF2	E3 ubiquitin-protein ligase RING2	3.27	1	1	55.3
P19474	475	54.1	6.4	TRIM21	E3 ubiquitin-protein ligase TRIM21	13.89	9	5	130.3
P19474-2	398	45.0	6.7	TRIM21	E3 ubiquitin-protein ligase TRIM21	10.05	6	3	3.0
Q5T4S7	5183	573.5	6.0	UBR4	E3 ubiquitin-protein ligase UBR4	3.2	13	9	216.3
O95071	2799	309.2	5.8	UBR5	E3 ubiquitin-protein ligase UBR5	3.07	10	4	97.1
G3GC05	363	39.9	4.4	Erk1/2	Erk1/2	19.32	8	2	54.1
Q14240	407	46.4	5.5	EIF4A2	Eukaryotic initiation factor 4A-II	28.75	14	2	273.8
P63241	154	16.8	5.2	EIF5A	Eukaryotic translation initiation factor 5A-1	20.13	5	3	90.4
Q9Y5B9	1047	119.8	5.7	SUPT16H	FACT complex subunit SPT16	4.78	5	4	23.5
Q9NVI1	1328	149.2	6.7	FANCI	Fanconi anemia group I protein	12.12	18	12	245.0
Q9UNN5	650	73.9	4.9	FAF1	FAS-associated factor 1	13.38	7	6	171.5
Q96AC1-2	633	72.4	7.5	FERMT2	Fermitin family homolog 2	7.42	2	2	35.1
Q9Y613	1164	126.5	6.4	FHOD1	FH1/FH2 domain-containing protein 1	5.58	9	5	183.0
Q5T1M5	1219	133.5	5.2	FKBP15	FK506-binding protein 15	6.48	8	5	154.3
P14635	433	48.3	7.5	CCNB1	G2/mitotic-specific cyclin-B1	16.17	5	4	119.2
Q9BSJ2	902	102.5	6.8	TUBGCP2	Gamma-tubulin complex component 2	13.75	14	9	246.9
Q12789	2109	238.7	7.3	GTF3C1	General transcription factor 3C polypeptide 1	2.28	3	2	64.6
Q9Y5Q9	886	101.2	5.1	GTF3C3	General transcription factor 3C polypeptide 3	5.42	5	3	112.0
Q9Y5Q8	519	59.5	6.9	GTF3C5	General transcription factor 3C polypeptide 5	5.01	3	2	79.1
Q9H4A5	285	32.7	5.8	GOLPH3L	Golgi phosphoprotein 3-like	5.26	2	1	49.5
Q7L5D6	327	36.5	5.4	GET4	Golgi to ER traffic protein 4 homolog	8.26	3	2	56.2
Q92538	1859	206.3	5.7	GBF1	Golgi-specific brefeldin A-resistance guanine nucleotide exchange factor 1	6.67	12	8	189.5
Q96CS2	278	31.8	5.5	HAUS1	HAUS augmin-like complex subunit 1	7.19	2	1	46.8
Q53T59	392	42.8	5.0	HS1BP3	HCLS1-binding protein 3	4.34	2	1	39.6

Q9H583	2144	242.2	6.5	HEATR1	HEAT repeat-containing protein 1	3.73	8	4	179.4
P08107	641	70.0	5.7	HSPA1A	Heat shock 70 kDa protein 1A/1B	29.95	26	11	310.4
P34932	840	94.3	5.2	HSPA4	Heat shock 70 kDa protein 4	3.93	5	3	81.8
P11142	646	70.9	5.5	HSPA8	Heat shock cognate 71 kDa protein	32.97	24	12	383.1
P11142-2	493	53.5	5.9	HSPA8	Heat shock cognate 71 kDa protein	24.14	20	6	21.6
O75506	76	8.5	4.4	HSBP1	Heat shock factor-binding protein 1	32.89	2	1	50.7
Q12931	704	80.1	8.2	TRAP1	Heat shock protein 75 kDa, mitochondrial	6.96	8	3	165.6
P04792	205	22.8	6.4	HSPB1	Heat shock protein beta-1	18.05	4	3	76.5
P07900	732	84.6	5.0	HSP90AA1	Heat shock protein HSP 90-alpha	41.94	45	12	710.5
P08238	724	83.2	5.0	HSP90AB1	Heat shock protein HSP 90-beta	41.3	50	11	866.2
P09651	372	38.7	9.1	HNRNPA1	Heterogeneous nuclear ribonucleoprotein A1	13.17	5	3	117.4
P09651-3	267	29.4	9.1	HNRNPA1	Heterogeneous nuclear ribonucleoprotein A1	21.35	14	4	9.1
P55795	449	49.2	6.3	HNRNPH2	Heterogeneous nuclear ribonucleoprotein H2	11.14	5	1	77.5
P31942-3	297	31.5	7.3	HNRNPH3	Heterogeneous nuclear ribonucleoprotein H3	15.82	6	3	37.0
O00422	153	17.5	9.3	SAP18	Histone deacetylase complex subunit SAP18	10.46	1	1	31.3
H0YFX9	92	10.0	9.7	H2AFJ	Histone H2A	20.65	1	1	44.1
P50502	369	41.3	5.3	ST13	Hsc70-interacting protein	13.28	7	4	163.5
Q16543	378	44.4	5.2	CDC37	Hsp90 co-chaperone Cdc37	10.58	6	3	90.8
P42858	3142	347.4	6.2	HTT	Huntingtin	0.73	1	1	37.1
O75146	1068	119.3	6.7	HIP1R	Huntingtin-interacting protein 1-related protein	7.96	10	7	226.6
Q27J81	1249	135.5	5.4	INF2	Inverted formin-2	4.08	5	3	111.9
Q15051	598	68.9	9.1	IQCB1	IQ calmodulin-binding motif-containing protein 1	6.69	4	3	111.8
Q96EK5	621	71.8	5.5	KIAA1279	KIF1-binding protein	4.51	3	2	112.4
Q8NBT2	197	22.4	4.7	SPC24	Kinetochore protein Spc24	26.4	5	4	105.6
Q03252	600	67.6	5.4	LMNB2	Lamin-B2	5.17	4	1	38.2
P46379	1132	119.3	5.6	BAG6	Large proline-rich protein BAG6	12.1	13	10	275.2
Q9P260	1216	134.5	5.5	KIAA1468	LisH domain and HEAT repeat-containing protein KIAA1468	3.78	4	3	88.1
Q9NRZ9	838	97.0	7.9	HELLS	Lymphoid-specific helicase	9.19	9	6	163.4
Q7LBC6	1761	191.5	7.2	KDM3B	Lysine-specific demethylase 3B	1.59	3	2	53.8
Q8IWC1	876	98.4	9.3	MAP7D3	MAP7 domain-containing protein 3	2.85	3	2	64.2
Q9NU22	5596	632.4	5.7	MDN1	Midasin	4.22	19	13	270.6
O15264	365	40.2	6.9	MAPK13	Mitogen-activated protein kinase 13	8.77	3	1	36.3
P61081	183	20.9	7.7	UBE2M	NEDD8-conjugating enzyme Ubc12	19.67	3	3	47.8
Q8NFP9	2946	327.6	6.2	NBEA	Neurobeachin	1.19	3	1	76.6
Q9UBB6	729	78.8	5.5	NCDN	Neurochondrin	1.92	2	1	98.2
Q8IXT1	998	111.5	7.2	NOXIN	Nitric oxide-inducible gene protein	1	1	1	35.3
Q00653	900	99.2	6.5	NFKB2	Nuclear factor NF-kappa-B p100 subunit	8.12	6	1	34.7
Q14980	2115	238.1	5.8	NUMA1	Nuclear mitotic apparatus protein 1	17.64	35	22	638.7
P57740	925	106.3	5.4	NUP107	Nuclear pore complex protein Nup107	12.65	10	7	218.9
Q92621	2012	227.8	6.2	NUP205	Nuclear pore complex protein Nup205	14.76	26	19	443.7
P37198	522	53.2	5.3	NUP62	Nuclear pore glycoprotein p62	15.9	7	5	110.0
Q9BVL2	599	60.9	9.3	NUPL1	Nucleoporin p58/p45	2.17	2	1	87.5
P22392	152	17.3	8.4	NME2	Nucleoside diphosphate kinase B	23.68	3	3	56.4
O00459	1358	149.4	8.1	PIK3R2	Phosphatidylinositol 3-kinase regulatory subunit beta	6.78	11	2	37.2
O00750	1634	179.8	7.1	PIK3C2B	Phosphatidylinositol 4-phosphate 3-kinase C2 domain-containing subunit beta	5.79	4	1	31.0
Q15149	4684	531.5	6.0	PLEC	Plectin	4.57	20	15	392.4

Q15149-7	4515	512.3	5.8	PLEC	Plectin	4.81	29	19	23.7
P02545	664	74.1	7.0	LMNA	Prelamin-A/C	46.08	72	26	33.5
Q14669	1992	220.3	8.5	TRIP12	Probable E3 ubiquitin-protein ligase TRIP12	1.46	4	2	117.2
Q86U06-4	405	44.5	8.7	RBM23	Probable RNA-binding protein 23	10.12	2	2	44.2
Q9BUL8	212	24.7	8.2	PDCD10	Programmed cell death protein 10	21.7	4	3	145.3
Q53EL6	469	51.7	5.2	PDCD4	Programmed cell death protein 4	7.04	3	2	102.8
O75340	191	21.9	5.4	PDCD6	Programmed cell death protein 6	6.81	1	1	67.9
Q8TCG1	905	102.1	6.2	KIAA1524	Protein CIP2A	8.07	9	6	149.3
Q9NSV4	1193	136.8	7.0	DIAPH3	Protein diaphanous homolog 3	4.02	6	3	132.9
Q9UJC3	728	84.6	5.1	HOOK1	Protein Hook homolog 1	1.65	2	1	78.6
P18583	2426	263.7	5.6	SON	Protein SON	2.51	8	4	147.9
O15027	2179	233.4	5.6	SEC16A	Protein transport protein Sec16A	1.51	3	3	60.3
A6NL28	223	26.3	4.5	N/A	Putative tropomyosin alpha-3 chain-like protein	10.31	11	2	147.3
B4DRT3	511	55.9	7.5	N/A	Pyruvate kinase	9.59	7	4	12.5
Q6P996	788	86.7	5.4	PDXDC1	Pyridoxal-dependent decarboxylase domain-containing protein 1	10.79	10	5	20.6
P14618	531	57.9	7.8	PKM2	Pyruvate kinase isozymes M1/M2	18.27	11	6	215.5
Q9Y3P9	1069	121.7	5.2	RABGAP1	Rab GTPase-activating protein 1	2.15	2	2	76.6
Q15042	981	110.5	5.6	RAB3GAP1	Rab3 GTPase-activating protein catalytic subunit	11.62	8	6	128.7
Q9H2M9	1393	155.9	5.6	RAB3GAP2	Rab3 GTPase-activating protein non-catalytic subunit	7.82	9	8	137.1
P31749	480	55.7	6.1	AKT1	RAC-alpha serine/threonine-protein kinase	20.21	10	7	188.5
P04049	648	71.4	9.3	RAF1	RAF proto-oncogene serine/threonine-protein kinase	9.23	3	2	49.9
Q9UN86	482	54.1	5.6	G3BP2	Ras GTPase-activating protein-binding protein 2	6.85	4	2	129.5
Q15404	277	31.5	8.6	RSU1	Ras suppressor protein 1	11.19	2	2	39.7
P61026	200	22.5	8.4	RAB10	Ras-related protein Rab-10	16.5	4	1	108.7
Q15907	218	24.5	5.9	RAB11B	Ras-related protein Rab-11B	32.11	8	4	186.8
P61106	215	23.9	6.2	RAB14	Ras-related protein Rab-14	23.72	6	3	155.7
P51149	207	23.5	6.7	RAB7A	Ras-related protein Rab-7a	35.27	7	6	92.5
P40938	356	40.5	8.3	RFC3	Replication factor C subunit 3	3.93	1	1	57.7
P40937	340	38.5	7.2	RFC5	Replication factor C subunit 5	13.82	6	3	86.0
Q68EM7	881	95.4	7.6	ARHGAP17	Rho GTPase-activating protein 17	2.84	3	2	81.6
Q92974	986	111.5	7.3	ARHGEF2	Rho guanine nucleotide exchange factor 2	7.2	8	5	214.5
Q9BV68	326	35.6	5.7	RNF126	RING finger protein 126	4.29	2	1	82.7
Q9UBF6	113	12.7	5.4	RNF7	RING-box protein 2	10.62	2	1	70.9
O43251	390	41.3	7.3	RBFOX2	RNA binding protein fox-1 homolog 2	3.85	2	1	61.4
Q9NRX1	252	27.9	9.7	PNO1	RNA-binding protein PNO1	5.56	2	1	55.4
Q5JTH9	1297	143.6	8.7	RRP12	RRP12-like protein	2.39	3	2	60.3
Q8WVM8	642	72.3	6.3	SCFD1	Sec1 family domain-containing protein 1	9.66	6	4	109.1
Q14674	2120	233.0	7.5	ESPL1	Separin	0.75	2	1	50.8
Q05519	484	53.5	10.5	SRSF11	Serine/arginine-rich splicing factor 11	3.51	2	1	40.5
P84103	164	19.3	11.6	SRSF3	Serine/arginine-rich splicing factor 3	14.02	2	1	54.6
O14757	476	54.4	8.3	CHEK1	Serine/threonine-protein kinase Chk1	3.15	2	1	69.9
Q9UPZ9	632	71.4	9.8	ICK	Serine/threonine-protein kinase ICK	2.85	2	1	47.6
Q7KZI7	788	87.9	9.7	MARK2	Serine/threonine-protein kinase MARK2	4.57	3	2	69.6
P42345	2549	288.7	7.2	MTOR	Serine/threonine-protein kinase mTOR	1.69	3	2	38.3
Q16513	984	112.0	6.3	PKN2	Serine/threonine-protein kinase N2	4.07	5	3	122.3
P51955-3	326	37.9	8.6	NEK2	Serine/threonine-protein kinase Nek2	3.68	2	1	25.1

P53350	603	68.2	8.9	PLK1	Serine/threonine-protein kinase PLK1	9.78	5	3	61.5
Q6PHR2	472	53.4	7.4	ULK3	Serine/threonine-protein kinase ULK3	2.97	2	1	51.7
Q9H4A3	2382	250.6	6.3	WNK1	Serine/threonine-protein kinase WNK1	1.81	6	4	148.1
P62714	309	35.6	5.4	PPP2CB	Serine/threonine-protein phosphatase 2A catalytic subunit beta isoform	10.36	2	2	49.1
O00743	305	35.1	5.7	PPP6C	Serine/threonine-protein phosphatase 6 catalytic subunit	12.13	4	3	81.6
Q96C92	435	47.9	5.1	SDCCAG3	Serologically defined colon cancer antigen 3	6.44	2	2	51.1
P58004	480	54.5	5.9	SESN2	Sestrin-2	3.33	1	1	65.0
P42224	750	87.3	6.0	STAT1	Signal transducer and activator of transcription 1-alpha/beta	1.73	2	1	46.8
P63165	101	11.5	5.5	SUMO1	Small ubiquitin-related modifier 1	6.93	1	1	32.4
P61956	95	10.8	5.5	SUMO2	Small ubiquitin-related modifier 2	12.63	1	1	46.0
O60493	162	18.8	8.7	SNX3	Sorting nexin-3	15.43	4	2	66.1
Q9UBP0-4	498	54.4	9.5	SPAST	Spastin	4.42	1	1	29.1
P63208	163	18.6	4.5	SKP1	S-phase kinase-associated protein 1	22.7	2	2	81.0
P16949	149	17.3	6.0	STMN1	Stathmin	8.72	1	1	54.9
Q92922	1105	122.8	5.8	SMARCC1	SWI/SNF complex subunit SMARCC1	1.36	2	1	58.7
O60264	1052	121.8	8.1	SMARCA5	SWI/SNF-related matrix-associated actin-dependent regulator of chromatin subfamily A member 5	4.94	6	3	135.2
Q9Y6J9	622	67.8	9.0	TAF6L	TAF6-like RNA polymerase II p300/CBP-associated factor-associated factor 65 kDa subunit 6L	3.7	1	1	59.6
O60784	492	53.8	4.7	TOM1	Target of Myb protein 1	5.69	3	2	93.0
Q86VP1-3	563	65.1	5.4	TAX1BP1	Tax1-binding protein 1	4.62	1	1	27.1
Q4KMP7	808	87.1	9.2	TBC1D10B	TBC1 domain family member 10B	2.6	1	1	39.2
Q66K14	1250	140.4	5.2	TBC1D9B	TBC1 domain family member 9B	6.4	8	5	119.4
P17987	556	60.3	6.1	TCP1	T-complex protein 1 subunit alpha	39.39	24	16	525.0
P78371	535	57.5	6.5	CCT2	T-complex protein 1 subunit beta	30.47	19	11	323.1
P50991	539	57.9	7.8	CCT4	T-complex protein 1 subunit delta	27.09	18	11	313.5
P48643	541	59.6	5.7	CCT5	T-complex protein 1 subunit epsilon	31.05	19	14	316.7
Q99832	543	59.3	7.6	CCT7	T-complex protein 1 subunit eta	30.94	20	12	356.9
P49368	545	60.5	6.5	CCT3	T-complex protein 1 subunit gamma	23.67	19	11	345.9
P50990	548	59.6	5.6	CCT8	T-complex protein 1 subunit theta	33.76	28	16	501.2
P40227	531	58.0	6.7	CCT6A	T-complex protein 1 subunit zeta	28.63	18	10	331.5
Q9Y4R8	837	91.7	5.8	TELO2	Telomere length regulation protein TEL2 homolog	2.99	4	2	84.6
Q5UIP0	2472	274.3	5.5	RIF1	Telomere-associated protein RIF1	9.75	20	14	287.9
Q15554	500	55.5	9.2	TERF2	Telomeric repeat-binding factor 2	2.8	1	1	26.1
Q9BZE9	553	60.1	6.6	ASPSCR1	Tether containing UBX domain for GLUT4	2.89	2	1	49.4
Q9BZE9-3	501	54.5	6.9	ASPSCR1	Tether containing UBX domain for GLUT4	18.76	20	7	49.6
O95801	387	44.7	5.6	TTC4	Tetratricopeptide repeat protein 4	12.92	4	2	104.7
P55072	806	89.3	5.3	VCP	Transitional endoplasmic reticulum ATPase	3.35	4	2	71.9
P49755	219	25.0	7.4	TMED10	Transmembrane emp24 domain-containing protein 10	12.33	3	2	110.9
P57088	247	28.0	9.7	TMEM33	Transmembrane protein 33	4.86	2	1	72.2
Q9Y5L0	923	104.1	5.6	TNPO3	Transportin-3	14.63	13	10	123.3
Q12888	1972	213.4	4.7	TP53BP1	Tumor suppressor p53-binding protein 1	1.06	1	1	48.2
Q99816	390	43.9	6.5	TSG101	Tumor susceptibility gene 101 protein	6.15	2	2	66.4
P41240	450	50.7	7.1	CSK	Tyrosine-protein kinase CSK	5.78	3	2	100.3
Q9H3S7	1636	178.9	6.9	PTPN23	Tyrosine-protein phosphatase non-receptor type 23	4.22	4	3	76.6
P43378	593	68.0	8.0	PTPN9	Tyrosine-protein phosphatase non-receptor type 9	2.19	2	1	88.8
Q9UMX0	589	62.5	5.1	UBQLN1	Ubiquilin-1	6.96	3	3	46.2
Q14694	798	87.1	5.3	USP10	Ubiquitin carboxyl-terminal hydrolase 10	10.03	6	5	87.2

P62979	156	18.0	9.6	RPS27A	Ubiquitin-40S ribosomal protein S27a	18.59	4	2	64.1
Q16763	222	23.8	8.4	UBE2S	Ubiquitin-conjugating enzyme E2 S	16.22	3	3	73.8
Q9GZZ9	404	44.8	4.8	UBA5	Ubiquitin-like modifier-activating enzyme 5	25.5	10	5	159.6
Q9BZV1-2	388	43.8	5.7	UBXN6	UBX domain-containing protein 6	4.9	1	1	26.4
Q6NW34	567	64.5	9.7	C3orf17	Uncharacterized protein C3orf17	2.47	2	1	44.2
Q5VIR6-4	832	94.3	6.7	VPS53	Vacuolar protein sorting-associated protein 53 homolog	6.49	3	3	36.5
O75396	215	24.6	6.9	SEC22B	Vesicle-trafficking protein SEC22b	18.14	4	3	68.3
Q00341	1268	141.4	6.9	HDLBP	Vigilin	13.41	16	3	274.2
P09327	827	92.6	6.4	VIL1	Villin-1	16.08	32	11	39.5
P08670	466	53.6	5.1	VIM	Vimentin	43.78	25	18	388.4
P18206	1134	123.7	5.7	VCL	Vinculin	2.12	4	2	77.9
Q15061	677	74.8	5.6	WDR43	WD repeat-containing protein 43	4.14	1	1	20.1
Q6UXN9	313	35.1	7.7	WDR82	WD repeat-containing protein 82	6.39	2	1	68.5
Q7Z5K2	1190	132.9	5.4	WAPAL	Wings apart-like protein homolog	6.55	9	5	152.4
Q96KR1	1074	116.9	9.0	ZFR	Zinc finger RNA-binding protein	1.58	2	1	97.3

[a]The Protein Coverage Summarizes can be used to determine the percent of the residues in each protein sequence that have been identified.

[b]Peptide Spectral Matches - An essential component of these algorithms is the scoring function used to evaluate the quality of peptide.

[c]The number of times for the peptide match.

[d]Ions score - If there are duplicate matches to the same peptide, then the lower scoring matches are shown in brackets.
