# Supplementary material for: VCP Phosphorylation-Dependent Interaction Partners Prevent Apoptosis in Helicobacter pylori-Infected Gastric Epithelial Cells
Source: PLoS One. 2013 Jan 31;8(1):e55724. doi: 10.1371/journal.pone.0055724 (PMC3561343; doi:10.1371/journal.pone.0055724)
Supplement: Table S3 — Top high-level functions identified by Ingenuity global function analysis of VCP-interacting proteins in H. pylori -infected AGS cells. (PDF) [file pone.0055724.s006.pdf]

Table S3. Top high-level functions identified by Ingenuity global function analysis of VCP-interacting proteins in *H. pylori*-infected AGS cells.

Function annotation	P value	Molecules
Cancer	8.26E-08	26s Proteasome, 14-3-3 protein, ACTB, AKT1, Alpha tubulin, BAX, Cofilin, COL1A2, Creb, Cyclin A, DDX17, DDX3X, EIF4B, Eif4g, ERK1/2, FLNA, Focal adhesion kinase, GNB2L1, Hdac, Histone h3, Histone h4, HNRNPK, Hsp27, Hsp70, Hsp90, HSP90AB1, HSPA1A/HSPA1B, HSPA8, IGF1R, Jnk, NFKB2, NPM1, MDM2, mTOR, P38 MAPK, p85 (pik3r), PI3K (complex), PDCD4, PLEC, Rac, Raf, Ras, RB1CC1, RPS6, TOP1, TP53, TUBA1A, TUBB, TUBB2C, Ubiquitin, VCP, VIM
Cell cycle progression	2.64E-07	26s Proteasome, 14-3-3 protein, AKT1, Ck2, Creb, Cyclin A, DDX17, ERK1/2, GNB2L1, Hdac, Histone h3, Histone h4, Hsp27, HSPA1A/HSPA1B, HSPA8, IGF1R, Jnk, NPM1, NUMA1, P38 MAPK, Pdgf Ab, PI3K (complex), Pka, PRMT5, Rac, Raf, Ras, RPS6, TOP1, TP53, TUBB
Death of cancer cells	3.41E-06	26s Proteasome, 14-3-3 protein, Actin, AKT1, Bax, Ck2, Creb, ERK1/2, FLNA, Focal adhesion kinase, GNB2L1, Hdac, HNRNPC, Hsp27, Hsp70, Hsp90, HSP90AB1, HSPA1A/HSPA1B, HSPA8, IGF1R, Jnk, NFKB2, NPM1, P38 MAPK, Pdgfr, PI3K (complex), Pka, PP2A, Ras, RB1CC1, TOP1, TP53, TUBA1A, Ubiquitin, VCP, YBX1
Growth of cells	2.97E-08	26s Proteasome, ACTB, AKT1, CAPZA1, COL1A2, Cyclin A, DDX3X, EIF4B, ERK1/2, Focal adhesion kinase, GNB2L1, Histone h3, Histone h4, HNRNPC, HNRNPK, HNRNPU, Hsp27, Hsp70, Hsp90, HSPA1A/HSPA1B, IGF1R, Jnk, NFKB2, NPM1, mTOR, P38 MAPK, PI3K (complex), Pka, PLEC, PP1 protein complex group, Rac, Ras, RB1CC1, TOP1, TP53, YBX1
Protein biosynthesis	1.01E-08	AKT1, DHX9, EIF4B, Eif4g, GNB2L1, HNRNPK, Jnk,P38 MAPK, PABPC1, PI3K (complex), RPL6, RPS6, TP53
Tumorigenesis	2.90E-06	14-3-3 protein, ACTB, AKT1, Bax , COL1A2, F Actin, FBL, FLNA, Focal adhesion kinase, Hdac, Histone H1, Histone h3, Histone h4, HNRNPU, Hsp27, Hsp70, HSPA1A/HSPA1B, HSPA1L, HSPA8, IGF1R, Jnk, NFKB2, NPM1, P38 MAPK, Pdgf Ab, PLEC, Profilin, RB1CC1, TOP1, TP53, TUBA1A, TUBB, TUBB2C, Ubiquitin, VCP, VIM
